# *Plasmodium vivax* antigen candidate prediction improves with the addition of *Plasmodium falciparum* data

**DOI:** 10.1038/s41540-024-00465-y

**Published:** 2024-11-13

**Authors:** Renee Ti Chou, Amed Ouattara, Shannon Takala-Harrison, Michael P. Cummings

**Affiliations:** 1 Center for Bioinformatics and Computational Biology, University of Maryland, College Park, College Park, MD USA; 2 Center for Vaccine Development and Global Health, University of Maryland School of Medicine, Baltimore, MD USA

**Keywords:** Immunology, Systems biology

## Abstract

Intensive malaria control and elimination efforts have led to substantial reductions in malaria incidence over the past two decades. However, the reduction in *Plasmodium falciparum* malaria cases has led to a species shift in some geographic areas, with *P. vivax* predominating in many areas outside of Africa. Despite its wide geographic distribution, *P. vivax* vaccine development has lagged far behind that for *P. falciparum*, in part due to the inability to cultivate *P. vivax* in vitro, hindering traditional approaches for antigen identification. In a prior study, we have used a positive-unlabeled random forest (PURF) machine learning approach to identify *P. falciparum* antigens based on features of known antigens for consideration in vaccine development efforts. Here we integrate systems data from *P. falciparum* (the better-studied species) to improve PURF models to predict potential *P. vivax* vaccine antigen candidates. We further show that inclusion of known antigens from the other species is critical for model performance, but the inclusion of only the unlabeled proteins from the other species can result in misdirection of the model toward predictors of species classification, rather than antigen identification. Beyond malaria, incorporating antigens from a closely related species may aid in vaccine development for emerging pathogens having few or no known antigens.

## Introduction

Malaria is an infectious disease caused by protozoan parasites of the *Plasmodium* genus, which exhibit a multi-staged, complex life cycle in the host and the vector^1^. Despite considerable reductions in the malaria burden over the past two decades, malaria incidence has plateaued, or even increased, in the past 5–7 years^2^. The hard-won progress is now in jeopardy due to the emergence of resistance in both the parasite and the vector^1–4^. Furthermore, with the reduction of *Plasmodium falciparum* in some endemic areas, a shift in species composition has been reported, with *Plasmodium vivax* predominating in many areas outside of Africa^5,6^. There are several factors likely contributing to this shift, including the ability of *P. vivax* to cause relapsing infections from dormant liver stages (hypnozoites), low parasite densities that can escape standard diagnostic tests, the early emergence of infective gametocytes prior to clinical symptom onset, as well as a shorter development cycle in the mosquito vector^7,8^. These factors will likely make elimination of *P. vivax* malaria more challenging than elimination of *P. falciparum* malaria^7^. Currently, there are only a few *P. vivax* vaccine candidates in the clinical development stage^8^. Vaccine development for *P. vivax* faces some similar challenges as *P. falciparum* vaccine development, including a complex parasite life cycle with multiple stages, where different antigens are expressed at each stage^9^, as well as genetic diversity, which is greater in *P. vivax* than in *P. falciparum*^10^. For immunogenic surface proteins, this genetic diversity may lead to vaccine escape, as observed in *P. falciparum*^11,12^. Additionally, the lack of in vitro culture capabilities for *P. vivax* further complicates the development of vaccines against this parasite^9^.

Systems data, including whole genome sequence data, has become increasingly available for many pathogens, including *Plasmodium*^13–15^. Leveraging these data, Rino Rappuoli and colleagues proposed a reverse vaccinology approach to identify potential vaccine antigen candidates targeting the B strains of *Neisseria meningitidis* (meningococci), which resulted in the licensed MenB vaccine^16,17^. Reverse vaccinology has been employed to a lesser extent to identify potential vaccine antigens in *Plasmodium* species^18^. However, these studies have primarily focused on *P. falciparum*, and the protein or epitope selection criteria have been limited^19,20^. Recently, we described a machine learning-based approach designed to learn the properties of a limited set of known antigens using a large number of protein variables^21^. The machine learning algorithm we used, known as positive-unlabeled random forest (PURF)^22^, is particularly useful for classification problems where some labels are missing, and thus, only a portion of the positive class is labeled^23,24^. In this recent study, we trained PURF on *P. falciparum* with a limited number of high-quality known antigens and prioritized top-ranking candidate antigens from the unlabeled proteins^21^. Positive-unlabeled learning has been applied to identify correlates of infection risk and protective immunity^25,26^, but to our knowledge, its use in the context of reverse vaccinology has been limited to our recent study on *P. falciparum*^21^.

**Fig. 1:**
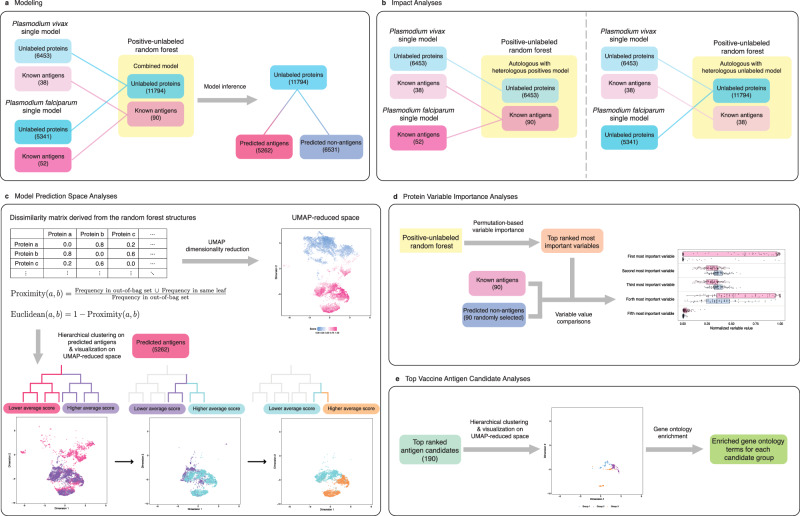
Schematic of the antigen identification analytical framework. **a** Modeling pipeline of *P. vivax* vaccine antigen identification enhanced by combining additional information learned from the *P. falciparum* antigen identification model. **b** Impact analysis to decipher how the additional data could improve the antigen identification pipeline. **c** Model prediction space analysis to understand the distributions of proteins in the prediction space. **d** Protein variable importance analysis to identify relevant variables impacting the prediction results. **e** Top vaccine antigen candidate analysis to characterize the biological functions of candidate groups.

Here, we utilize the PURF algorithm to train a machine learning model to identify potential vaccine antigen candidates for *P. vivax* (Fig. 1). Recognizing that systems data for a single species are limited (e.g., the number proteins in a proteome is small), and known antigens are even more limited still, we further improve the model accuracy by adding data from *P. falciparum*, hereafter also referred to as heterologous data (Fig. 1a). The impact of incorporating the heterologous data is then analyzed based on two data types: heterologous known antigens and heterologous unlabeled proteins. Our results demonstrate that the inclusion of known antigens from a different species improves the accuracy of vaccine antigen predictions. However, the integration of only the unlabeled proteins from another species could inadvertently amplify effects related to species distinction, potentially misdirecting the classification algorithm to focus more on protein variables that differentiate the two species over the task of antigen identification. Thus, it is critical to include labeled data from another species in the final model. To understand variables that are important for *P. vivax* antigen identification, we then conduct variable importance analysis on the final model. Top-ranking candidate antigens are clustered into three groups, for which we provide further characterization. Our methodology demonstrates potential for prioritizing and accelerating malaria vaccine development for *P. vivax* and other minority *Plasmodium* species, presenting a promising solution for addressing the global burden of malaria.

## Results

### Data engineering and model training

**Fig. 2:**
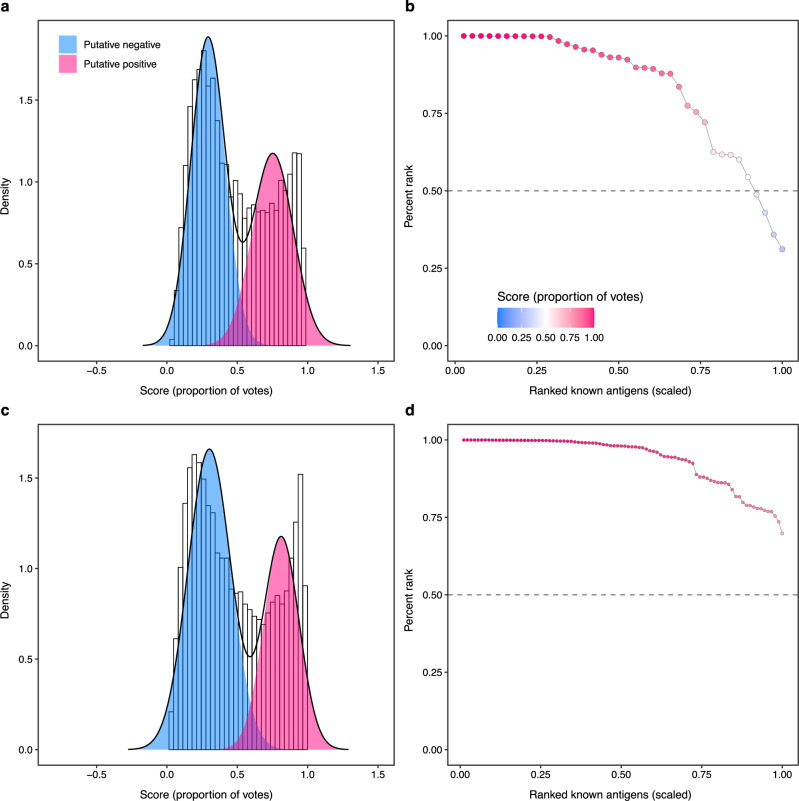
Performance of PURF models with the optimal hyper-parameter setting. **a, c** Probability score (proportion of votes) distributions of the *P. vivax* model (**a**) and the combined model (**b**). The magenta and blue shaded areas are respectively putative positives (antigens) and putative negatives (non-antigens). The black curve was computed using a two-component Gaussian mixture model. The areas under the receiver operating characteristic curves (AUROC) for the *P. vivax* and combined models were 0.99 and 0.995, respectively. **b, d** Evaluation of known antigen scores of the *P. vivax* (**b**) and the combined model (**d**). Dots represent known antigens. The *x*-axis shows scaled ranks of the known antigens, and the *y*-axis denotes percentile ranks (the higher the better) of the known antigens across all proteins in the data set. The respective areas under the curves for the *P. vivax* and the combined models were 0.84 and 0.94.

*P. vivax* protein variables were derived from publicly available genome assemblies, as well as various bioinformatics analyses, including genomic, immunological, proteomic, and structural data types (refer to Supplementary Data File 1 and Methods for further details). In this study, our existing database^21^ containing solely *P. falciparum* protein variables was expanded to include data from *P. vivax*. The data set contains 6491 *P. vivax* proteins and 272 protein variables (Supplementary Data File 2). The genomic variables include measurements obtained from single nucleotide polymorphism (SNP) analysis; the immunological variables contain T-cell, B-cell, and other types of epitopes summarized at the protein level; the proteomic variables include subcellular localization, glycosylation sites, and physiochemical properties; the structural variables comprise information about sequence complexity, transmembrane helices, and surface accessibility. The selection of antigen labels for training the machine learning models was based on the union set of antigens identified in the literature and those identified using the immune epitope database (IEDB)^27^, resulting in 38 known *P. vivax* antigens labeled as positives, with remaining proteins designated as unlabeled (Supplementary Table 1). The amount of systems data for a single species is inherently limited, because of the small, finite number of genes, proteins, and metabolites within a species. This limitation is especially acute in the study of antigens, because of a paucity of experimentally validated antigenic proteins. We therefore seek to overcome this inherent limitation by adding data from another species, *P. falciparum*. Thus, we consider two types of homologous data. The first are autologous, those data from within the same species, and the second are heterologous, those data from another species. Furthermore, in the context of using positive-unlabeled learning to predict possible antigenic proteins, both autologous and heterologous data are subdivided in two types: known antigens and unlabeled proteins (Fig. 1a). Positive-unlabeled random forest (PURF)^22^ models with different hyper-parameter settings were constructed for the *P. vivax* data (Supplementary Data File 3). Specifically, we compared models having different positive levels representing the prior proportion of potential antigens within the whole proteome. Positive-unlabeled learning can be viewed as a special case of semi-supervised learning, where the positives and negatives in the unlabeled data are predicted from learning the labeled data and the underlying structures in the unlabeled data^23^. Model performance was assessed using three metrics: the area under the receiver operating characteristic curve (AUROC), which measures the separation of antigen and non-antigens based on prediction scores; the area under the curve (AUC) for percentile ranks of known antigens, quantifying how these known antigens are ranked amongst all proteins; and a metric similar to the *F*_*1*_ score, which accounts for both recall and precision (see Methods). As the research goal was to identify potential vaccine antigens for *P. vivax*, evaluation scores were computed from out-of-bag samples and were used relatively to select the model with the optimal hyper-parameter setting, but not as an estimation of generalization to unseen data from other species. The evaluation of the predicted probability score distributions showed that the *P. vivax* model with a positive level of 0.5 had the highest AUROC, 0.99, indicating the model capability in identifying antigens from non-antigens (Supplementary Fig. 1). Further investigation of known antigen predictions revealed that the model, with positive level of 0.5, identified 34 known antigens with percentile ranks exceeding 0.5, and a moderately high AUC of 0.84, showing the model was able to identify known antigens. The explicit positive recall (EPR; percentage of correctly predicted labeled positives)^24,28^ for known antigen prediction accuracy was 87% (Supplementary Fig. 2 and Table 1). The *P. falciparum* data were then added into the training data set to explore whether these data would improve the current model. Thus, PURF models were trained using a combined data set including data from both species (Supplementary Data File 4, 5). The combined model had an AUROC of 0.995 based on the probability score distribution, higher than that of the model including *P. vivax* data only (Fig. 2a, b and Supplementary Fig. 3). All 90 known antigens (38 *P. vivax*, 52 *P. falciparum*) were correctly predicted (EPR = 100%) by the combined model (Table 1). All known antigens had percentile ranks above 0.5 across the entire combined protein set, with an AUC of 0.94 (Supplementary Fig. 4 and Fig. 2b, d). Moreover, the alternative metric of the *F*_*1*_ score has values of 1.71 for the *P. vivax* model and 2.22 for the combined model, suggesting an improvement in antigen predictions for both species.

**Table 1:** *P. vivax* and *P. falciparum* known antigen prediction accuracies of PURF models trained separately on *P. vivax*, *P. falciparum*, and combined data sets

PURF model	Prediction accuracy of *P. vivax* known antigens	Prediction accuracy of *P. falciparum* known antigens
***P. vivax*** **model**	33 / 38 = 0.87	47 / 52 = 0.90
***P. falciparum*** **model**	34 / 38 = 0.89	49 / 52 = 0.94
**Combined model**	38 / 38 = 1.00	52 / 52 = 1.00

### Comparison of single-species models and the combined model

As mentioned previously, we defined the data from the focus species as autologous and referred to the data from the other species as heterologous. We further compared the *P. vivax and P. falciparum* single-species models trained on the individual species data sets against the combined model by making heterologous predictions based on the single-species models. The *P. falciparum* single-species model accurately identified 89% of the heterologous *P. vivax* known antigens, and the *P. vivax* single-species model correctly predicted 90% of the heterologous *P. falciparum* known antigens. The combined species model predicted all *P. falciparum* and *P. vivax* known antigens correctly, resulting in a 100% accuracy (Table 1). We further focused on the antigen prediction results of the two single-species models to assess the prediction performance of merely merging the outputs of both models, instead of training on a combined data set. For *P. falciparum* known antigen predictions, the *P. falciparum* and *P. vivax* models together correctly identified all 52 antigens. However, for the *P. vivax* known antigens, only 35 out of 38 antigens were detected across both single-species models. To validate the single and combined models, we conducted an iterative validation process involving adversarial control^27^, where we removed one antigen label at a time and trained the model and computed the probability scores for the remaining known antigens using the trained model^21^. Aligning with the research goal of *P. vivax* vaccine antigen identification based on predicting the potential antigens and non-antigens from the unlabeled proteins, the adversarial control analysis was performed to more rigorously and accurately measure the model performance and robustness. Specifically, the mean difference in scores calculated by subtracting original model score from the score from the adversarial model, were between ±0.1 for both single-species models and the combined model (Supplementary Fig. 5). We observed two modes in the score difference distributions of the *P. vivax* and the combined models. To understand the underlying factors influencing the bimodal distribution, we examined the association between the two distribution modes and either one of the antigen sources, including labeling source (literature, IEDB, or both) and species type (*P. vivax* or *P. falciparum*; applicable to the combined model only) by compiling contingency tables and calculating odds ratios. The results indicated that there was a significant association between the two distribution modes and labeling source, for the *P. vivax* model (*p*-value = 2.60 × 10^−4^) and the combined model (*p*-value = 1.18 × 10^−5^). However, there was no significant association found between the two modes and species type (*p*-value = 0.66), suggesting that the model is robust to labeling of different species. Furthermore, the adversarial control experiments showed predicted mean accuracies of 94%, 90%, and 89% for identification of known antigens based on the *P. vivax*, *P. falciparum*, and combined models, respectively, suggesting the robustness of the antigen prediction results.

### Impacts of heterologous positives and unlabeled proteins on combined model performance

**Fig. 3:**
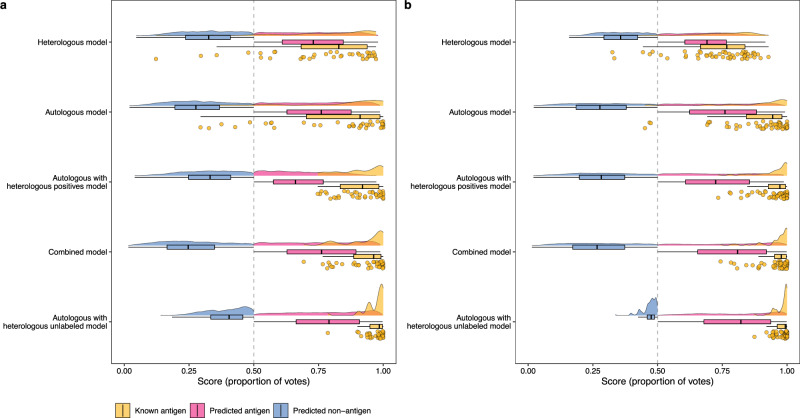
Probability score distributions of PURF models. Plots showing the score distributions for *P. vivax* (**a**) and *P. falciparum* (**b**) proteins. The results were generated from PURF models trained on different combinations of autologous and heterologous data. The amber, magenta, and blue colors represent known antigens, predicted antigens, and predicted non-antigens, respectively. Grey vertical dashed lines show the probability score of 0.5. Boxplots show the median with first and third quartiles, with whiskers denoting the extension of the 1.5 interquartile range from the first and third quartiles.

**Fig. 4:**
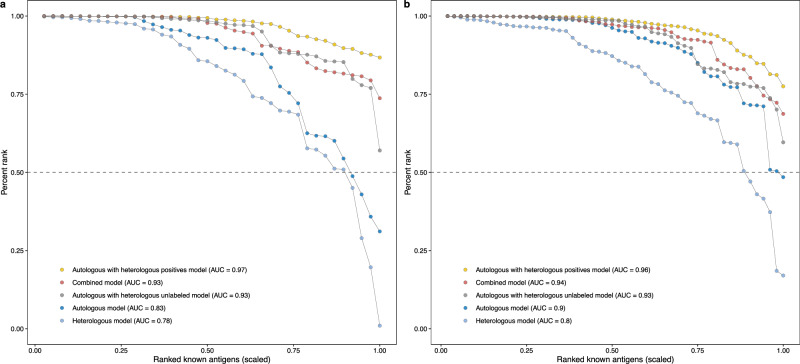
Evaluation of known antigen predictions of PURF models. Line plots showing the percentile ranks of known antigens in the sets of *P. vivax* (**a**) and *P. falciparum* (**b**) proteins. Dots represent known antigens, which are connected by lines indicating antigen predictions from PURF models trained on different combinations of autologous and heterologous data. The *x*-axis shows the scaled ranks of the known antigens only, and the *y*-axis indicates the percentile ranks (the higher the better) of the known antigens across the entire *P. vivax* (**a**) or *P. falciparum* (**b**) data sets. The areas under the curves (AUC) are noted in the legend text. The grey horizontal dashed lines indicate the percentile rank of 0.5.

To explore how the addition of *P. falciparum* data improved the accuracy of prediction of both *P. vivax* and *P. falciparum* antigens in the combined model, we further investigated the individual impacts of the positive and unlabeled proteins (Fig. 1b). In addition to the autologous single-species models and their predictions for heterologous proteins mentioned in the previous section, models were trained by either incorporating heterologous positives or heterologous unlabeled proteins (Fig. 1b, Table 2). The probability score distributions of *P. vivax* and *P. falciparum* proteins were analyzed separately for PURF models that were trained using different combinations of autologous and heterologous data. Compared to autologous and heterologous model predictions, the autologous model with heterologous positives and autologous model with heterologous unlabeled proteins predicted all known antigens correctly (EPR = 1) for both species (Fig. 3). However, among these models, the combined model had the largest difference in medians between the predicted antigen and non-antigen groups (Fig. 3), indicating the ability of the model to distinguish antigens more clearly from non-antigens among the unlabeled proteins. Moreover, it was observed that in the autologous models including heterologous unlabeled proteins, the predicted autologous non-antigens consistently received higher prediction scores compared to other models, suggesting a possible confounding of antigen identification and species classification (Fig. 3). Next, we focused on the labeled positives (known antigens) and the unlabeled proteins for each species. The percentile ranks of the known antigen predictions from the combined model, the autologous model with heterologous positives, and the autologous model with heterologous unlabeled proteins all had AUCs values > 0.9 for both species (Fig. 4). Antigen predictions of unlabeled proteins from both species (antigen or non-antigen) were further analyzed for their association with their corresponding species (*P. vivax* or *P. falciparum*). When using Cramér’s V to assess the strength of the association with species, we found that the single-species and combined models had a relatively weak association, <0.10. In contrast, the autologous model with heterologous positives had a strong association with species ( > 0.61), and the autologous model with heterologous unlabeled proteins displayed an even stronger association above 0.80 (Supplementary Table 2). Together with the observed score distributions of predicted antigens and non-antigens described above suggests that solely adding the heterologous unlabeled proteins from another species may misdirect the model to classify species rather than antigens. Additionally, it was noted that there is a significant relationship (*F*-statistic test; *p*-value = 0.012) between the mean tree depth in the model and the proportion of positives in the training data set (Supplementary Fig. 6), suggesting labeled data are important for a model to learn the comprehensive patterns among the data.

**Table 2:** Different combinations of data from *P. vivax* and *P. falciparum* and their corresponding model types

Autologous data	Heterologous data	Model type	Note
**All** ***P. vivax*** **proteins**	NA	Autologous model	Heterologous model with respect to *P. falciparum*
**All** ***P. vivax*** **proteins**	*P. falciparum* known antigens	Autologous with heterologous positives model	
**All** ***P. vivax*** **proteins**	*P. falciparum* unlabeled proteins	Autologous with heterologous unlabeled model	
**All** ***P. falciparum*** **proteins**	NA	Autologous model	Heterologous model with respect to *P. vivax*
**All** ***P. falciparum*** **proteins**	*P. vivax* known antigens	Autologous with heterologous positives model	
**All** ***P. falciparum*** **proteins**	*P. vivax* unlabeled proteins	Autologous with heterologous unlabeled model	
**Proteins from both species**	NA	Combined model

### Analysis of model prediction space and species effect

**Fig. 5:**
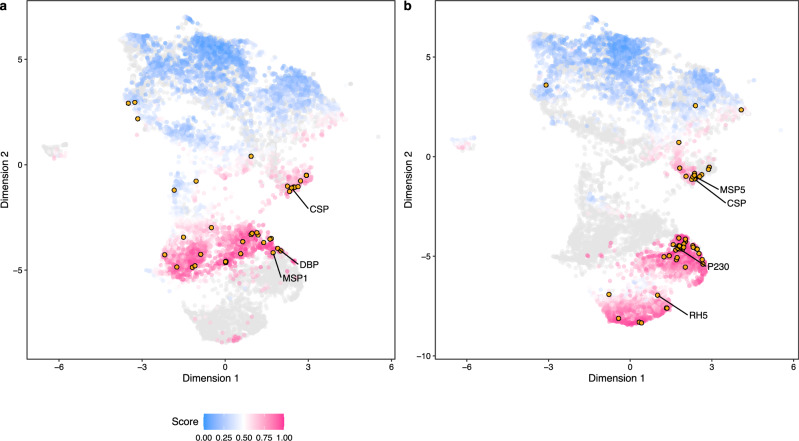
Visualization of the prediction space of the combined PURF model. Uniform manifold approximation and projection (UAMP) plots highlighting the *P. vivax* (**a**) and *P. falciparum* (**b**) proteins in the prediction space derived from the Euclidean distance matrix of the combined PURF model. Dots represent proteins in the combined set with *P. vivax* and *P. falciparum* data. Autologous proteins with higher probability scores are shown by darker magenta color and lower scores by darker blue color. Grey dots display *P. vivax* and *P. falciparum* heterologous proteins in (**a**) and (**b**), respectively. Dots with amber color indicate known antigens for *P. vivax* and *P. falciparum* respectively in (**a**) and (**b**), with corresponding reference antigens annotated by protein names.

To gain insights into the antigen prediction of the combined model, we computed the prediction space derived from the tree-based structures within the PURF model (Fig. 1c). The subsequent visualization revealed distinct clusters of predicted antigens for each species, whereas the predicted non-antigens appeared within a single, larger cluster irrespective of species (Fig. 5). To elucidate the effect of species on the predicted antigens, we conducted hierarchical clustering of the predicted antigens (Fig. 1c). Starting from the root of the dendrogram, the predicted antigens were iteratively divided into two groups, and the group with the higher mean predicted probability score was selected for the next iteration and continued for four iterations (Supplementary Fig. 7). For the first three iterations, the statistical analysis showed a significant association between the two clustering groups and species (*χ*^2^ test, *p*-values for the four iterations: 1.11 × 10^−10^, 6.50 × 10^−46^, <2.23 × 10^−308^, and 0.33). Notably, the third iteration had the strongest association between cluster group and species (Cramér’s V = 0.94; 95% CI: 0.93, 0.95), whereas the remaining three iterations demonstrated weaker association strengths (Cramér’s V for the first, second, and fourth iterations: 0.09, 0.24, and 0.02). The results indicated that there might be species-specific effects, with predicted antigens having higher scores. Thus, species-specific antigens may be identified with a higher score threshold. To gain a deeper understanding of whether the observed species effects were attributable to differences in amino acid composition of protein sequences in each species, we performed an association analysis between the amino acid frequencies and species. The results, based on Cramér’s V, showed there was a weak association of 0.20 across all proteins, with a slight increase in association strength to 0.24 for predicted antigens, and a lower association strength of 0.10 for predicted non-antigens, suggesting that amino acid composition may not be the primary driver of the observed species effect. Additionally, 84 *P. falciparum* known antigen orthologs in *P. vivax* were compared against a set of 84 top *P. vivax* candidate antigens identified from the combined model. The prediction scores from the *P. vivax* and combined model were significantly different for both the orthologs and top candidates (Supplementary Fig. 8), suggesting the addition of *P. falciparum* antigens improves antigen identification that can be generalized to non-orthologs.

### Variables contributing to *Plasmodium* antigen prediction

**Fig. 6:**
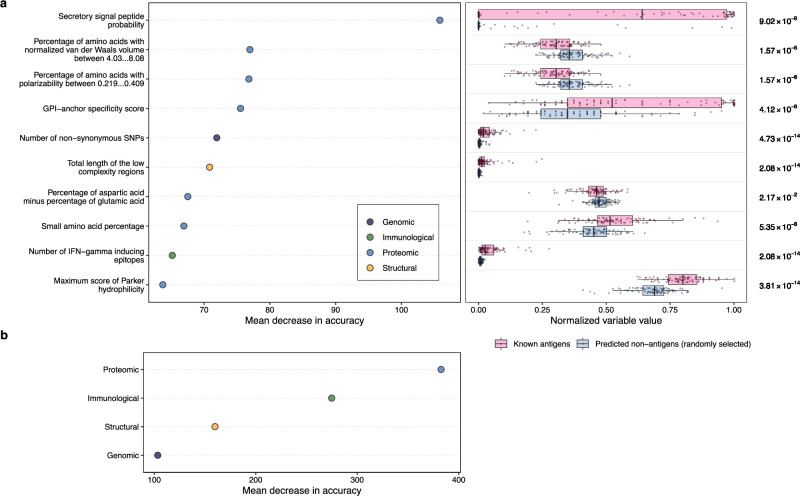
Model interpretation of the combined PURF model on the prediction of known antigens. **a** Top 10 important variables computed using permutation-based variable importance analysis on the tree-based PURF model. The left panel shows variable importance values in terms of mean decrease in prediction accuracy after variable permutation. The accuracy was calculated for the 90 known antigens from both *Plasmodium* species. Variables are categorized into genomic (dark blue), immunological (green), proteomic (blue), and structural (amber) data types. The right panel displays corresponding variable values normalized to range between 0 and 1. Magenta dots represent the 90 known antigens, and the blue dots show 90 predicted non-antigens that were randomly selected. Boxplots convey median with first and third quartiles, and the whiskers indicate the 1.5 interquartile range extended from the first and third quartiles. Two-sided Mann–Whitney tests were conducted with *p*-values adjusted using the Benjamini–Hochberg procedure, and *p*-values are shown on the right of the panel. **b** Permutation-based group variable importance analysis. Variables were grouped by data types and permuted together to calculate the mean decrease in accuracy across all trees in the model.

**Fig. 7:**
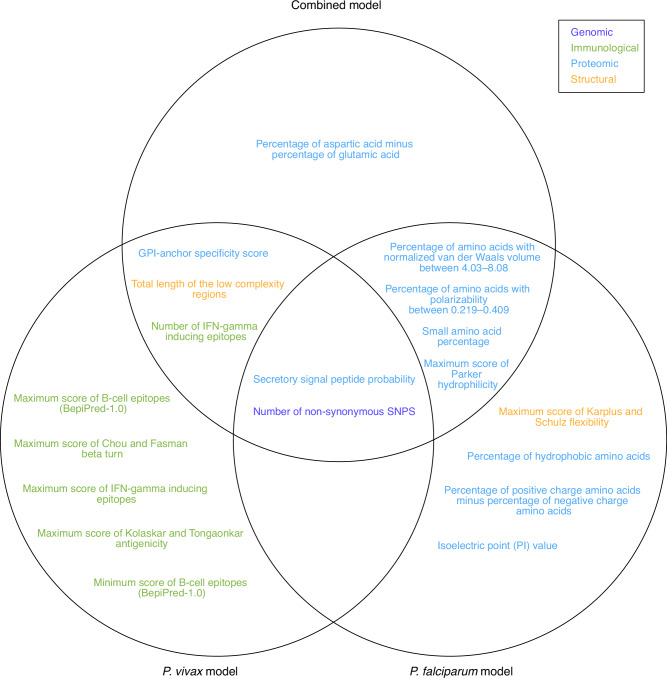
Venn diagram of top 10 important variables from different PURF models. Top important variables were identified separately from the combined, *P. vivax*, and *P. falciparum* models. Variable names are colored by the corresponding categories.

To understand the most important variables for *Plasmodium* antigen prediction using the combined models, we conducted a permutation-based variable importance analysis^29^ (Fig. 1d). Variables values from known antigens and predicted non-antigens were compared for the top important variables to impart biological insights (Fig. 1d). All four data types, including genomic, immunological, proteomic, and structural data, were represented in the top 10 most important variables (Fig. 6a). Among these 10 variables, the following exhibited higher values in known antigens compared to randomly selected non-antigens: secretory signal peptide probability, glycosylphosphatidylinositol (GPI)-anchor specificity score, number of non-synonymous single nucleotide polymorphisms (SNPs), total length of low complexity regions, small amino acid percentage, number of interferon (IFN)-gamma inducing epitopes, and maximum score of Parker hydrophilicity for predicted epitopes (Fig. 6a). In contrast, known antigens had a decreased percentage of amino acids with high normalized van der Waals volume (between 4.03–8.08), and a reduced percentage of amino acids with high polarizability (between 0.219–0.409) (Fig. 6a). The analysis of variable importance in the *P. vivax* single-species model showed that among the top 10 variables, five were immunological variables associated with B-cell epitopes, IFN-inducing epitopes, and antigenicity predictions (Supplementary Fig. 9a). Further analysis of the group variable importance, categorized by data types, indicated that proteomic and immunological variables may contribute more to the accuracy of antigen predictions in the combined model (Fig. 6b), which was consistent with the *P. vivax* group variable importance analysis results (Supplementary Fig. 9b). The comparison of top 10 important variables independently identified from the two single-species models and the combined model revealed that two variables, namely secretory signal peptide probability and number of non-synonymous SNPs, were identified by all three models (Fig. 7). Both the *P. vivax* and the combined model showed concordance on three additional variables, and the *P. falciparum* and the combined model showed agreement on an additional four variables (Fig. 7). We performed comparative analysis to examine the changes in importance values for the top 10 variables determined by the combined model. The results showed that in the combined model, all 10 variables had greater importance values compared to the two single-species models, suggesting the high influence of these variables regarding prediction accuracy in the combined model. When comparing the two single-species models, six variables had higher importance in *P. vivax*, and four variables demonstrated a higher importance value in *P. falciparum* (Supplementary Fig. 10), indicating the contributions of both species in the combined model.

### Characterization of top vaccine antigen candidates

**Fig. 8:**
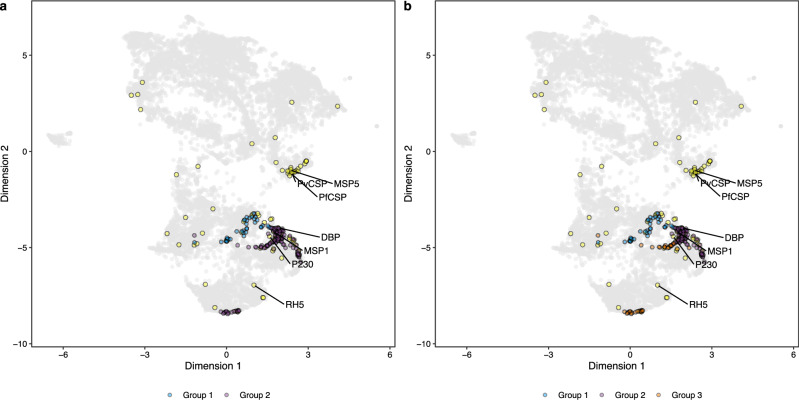
Clustering analysis of top candidate antigens. Hierarchical clustering was performed on the top candidate Euclidean distance matrix derived from the combined PURF model. The dendrogram was cut into two (**a**) and three (**b**) groups and visualized on uniform manifold approximation and projection (UMAP) plots computed from the Euclidean distance matrix of the combined *P. vivax* and *P. falciparum* proteomes. **a** Blue and purple dots respectively represent group 1 and group 2 candidate antigens when cutting the dendrogram into two groups. **b** Blue, purple, and orange dots show group 1, group 2, and group 3 candidate antigens, respectively, when generating three groups from the dendrogram. Yellow dots are known antigens from both *Plasmodium* species with protein names of reference antigens annotated. Grey dots are other unlabeled proteins from both species.1.

From the combined model, 190 top candidate antigens were selected based on their probability scores, where the score threshold was set above the median ranking of the 90 known antigens labeled positive in the combined PURF model. The top candidates comprised 35 proteins from *P. vivax* and 145 proteins from *P. falciparum*. We performed hierarchical clustering analysis to further characterize the top candidates (Fig. 1e). The Silhouette^30^ method identified two distinct groups, and the Elbow (or total within sum of square) method identified three groups in the dendrogram. We further visualized the groups in the prediction space and found that one of the two groups identified by the Silhouette method exclusively consisted of 35 candidate antigens from *P. vivax* (Fig. 8a, group 1 in blue). The other group was further divided into two based on the Elbow method, and one subcluster contains one candidate antigen from *P. vivax* and 38 candidate antigens from *P. falciparum* (Fig. 8b, group 3 in orange). Gene ontology (GO) enrichment analysis was performed to identify significantly enriched GO terms within the three clusters (Fig. 1e). Group 1, containing only *P. vivax* candidate antigens, was associated with intracellular activities and locations (Supplementary Fig. 11a). Group 2, comprising nine *P. vivax* and 107 *P. falciparum* antigens, had enriched GO terms related to immunological processes, host-pathogen interactions, and cell membranes (Supplementary Fig. 11b). Group 3 was characterized by its association with integral components of the membrane as well as the nucleus (Supplementary Fig. 11c). Moreover, there were five and four *P. vivax* candidates respectively in group 1 and group 2 not having orthologs in *P. falciparum*, indicating potential candidates for species-specific vaccine. A summary table (https://mrp-bioinformatics.github.io/malaria_antigen_candidates/) was generated for the top candidate antigens, providing detailed information regarding gene products, the closest known antigens, and the respective species corresponding to the known antigens (Supplementary Data File 6). Interestingly, for the nine *P. vivax* candidates in group 2, six were closest in the variable space to known antigens from *P. falciparum*, suggesting that the inclusion of *P. falciparum* data aided in the identification of potential vaccine candidate antigens for *P. vivax*.

## Discussion

Proteomes are limited in size, and this is often the case in other machine learning problems. The inclusion of external data, such as crowdsourcing and synthetic data generation, has become one of the strategies to improve machine learning models^31^. Using data from an additional species provides an opportunity to increase data set size using empirical data. Here, we showed that augmenting the *P. vivax* training data set with the *P. falciparum* proteome assisted in identification of antigens for both species. We further decomposed the impact of adding heterologous species data with the inclusion of either known antigens or unlabeled proteins. We demonstrated that inclusion of both heterologous known antigens and unlabeled proteins is important in improving the model performance. We also noticed that adding the heterologous unlabeled proteins alone may potentially transform the antigen identification problem into a species classification problem, leading to identification of proteins based on variables relevant to differentiating between *P. vivax* and *P. falciparum* rather than identification of proteins representing antigens from either species. We conducted a variable importance analysis to understand which protein variables have the greatest influence on antigen identification in the final combined model and observed that variables related to secretory signal peptides and GPI-anchors, hallmarks of surface proteins exposed to the host immune response, were the best predictors of antigens. Candidate antigens with the highest prediction scores clustered into three groups corresponding to intracellular activities, immune responses, and cellular membrane along with microtubules, respectively. Moreover, one of the closest reference antigens to group 1 is *P. vivax* MSP1, and *P. falciparum* P230 is the closest reference antigen to all three groups (Supplementary Data File 6).

Although *P. falciparum* is responsible for most malaria cases and deaths, *P. vivax* is the most geographically pervasive species, predominating in regions of Southeast Asia and South America. This wider geographic distribution and the unique biological properties of *P. vivax* that hinder elimination highlight the need for vaccines against this species. Most vaccine development efforts have focused on *P. falciparum* and have led to two WHO-recommended vaccines (i.e., Mosquirix^TM^ (RTS,S/AS01)^32^) and R21/MatrixM^33^. However, challenges remain for vaccine development against other human malaria parasites, such as *P. vivax*. To date, there have been only a few *P. vivax* vaccine candidates evaluated in clinical trials, including those targeting the circumsporozoite protein (PvCSP), Duffy-binding protein (PvDBP), and ookinete surface protein (Pvs25)^34^. Moreover, *P. vivax* cannot be maintained in continuous in vitro culture, posing a challenge for identification and preclinical evaluation of vaccine candidates^35^. Reverse vaccinology has been applied to malaria vaccine development to identify potential antigens or epitopes, with most studies focusing on *P. falciparum*^19,20,36,37^. One study of *P. vivax* performed computational analyses to identify B-cell epitopes in the merozoite surface protein-9 only^38^. Similarly, in another study, only a set of 39 *P. vivax* merozoite proteins were investigated, however, the selection criteria in terms of protein properties were not explicitly specified^39^. By employing a machine learning-based reverse vaccinology approach, we explored the whole proteome of *P. vivax* and many protein properties. By including data from *P. falciparum*, we improved the single-species model trained solely on *P. vivax* data and identified potential *P. vivax* vaccine antigen candidates in silico. Although future work would include experimental validation of the top antigen candidates, our approach helped facilitate the selection of candidate antigens compared to traditional approaches that could be time-consuming. To our knowledge, none of the *P. vivax* candidate antigens in group 1 have been identified before, and only two out of nine *P. vivax* candidate antigens in group 2 were discussed in the literature, with one being a potential invasion-related ligand (PVP01_0534300)^40^ and the other predicted as a secretory protein (PVP01_0948700)^41^. Most of the remaining unknown *P. vivax* antigen candidates are mostly annotated as putative proteins, with functions related to accessibility to the host immune system, such as TRAP-like protein, surface protein P113.

In this study, various bioinformatics tools have been developed to compute protein properties, which are essential in reverse vaccinology^42^. To be recognized by the host immune system, it is critical to include indicators showing that the proteins are exposed to the extracellular environment^18,42^. In our study, among all 272 protein variables, secretory signal peptide probability, computed using SignalP^43^, was ranked as the most important variable in model prediction for the combined and single species models. The results also showed the importance of non-synonymous SNPs in antigen prediction. Such non-synonymous polymorphisms are common in highly immunogenic, known antigens that have evolved diversity under immune pressure. Although non-synonymous SNPs are important predictors of antigens based on the known antigens used to train the models, genetic diversity can also contribute to vaccine escape, which has posed a problem for malaria vaccines^12^ and vaccines against other pathogens. Thus, further filtration of the predicted antigens can be applied to obtain potentially less immunogenic, but more genetically conserved candidate antigens, whose immunogenicity may be improved using an adjuvant. The *P. vivax* model in this study also identified more immunological variables among the most important predictor variables compared to the *P. falciparum* model. This result could stem from differences in amino acid composition between the species and the extremely AT-biased *P. falciparum* genome^44^, possibly affecting the quality of epitope predictions. Finally, for the antigen groups containing *P. vivax* antigens, group 1 was associated with intracellular activities, and group 2 proteins had gene ontology terms consistent with protein exposure to the immune system, including host cell plasma membrane, infected host cell surface knob, and integral component of membrane. As antigens need to be exposed to be recognized by the immune system^13,45^, *P. vivax* proteins in group 2 may be better candidates for inclusion in a vaccine. Additionally, there were four *P. vivax* proteins in group 2 without corresponding *P. falciparum* orthologs, indicating the model did not identify antigens based purely on protein sequence homology, as well as demonstrating the selection of potential species-specific vaccine targets.

In this study, data from a well-characterized species, *P. falciparum*, was included in machine learning models to inform vaccine antigen identification of a less well-characterized species, *P. vivax*, with reference antigens from both species being utilized to instruct the selection of *P. vivax* candidate antigens. The approach described here identified and prioritized candidate antigens from the *P. vivax* proteome, of which about 78.9% are proteins with putative or unknown functions. In addition to *P. falciparum* and *P. vivax*, clinical malaria cases have been reported for *P. ovale* and *P. malariae*^46–49^, and more recently, *P. knowlesi*, a simian *Plasmodium* species that causing an increased incidence of human clinical infections in Malaysia and areas of Southeast Asia^50–53^. The genomes of these minor *Plasmodium* species have also been sequenced, and the machine learning-based analytical methodology developed in this study can also be applied to identify vaccine candidate antigens for these species. Beyond malaria, our approach can be applied to other emerging pathogens having few known antigens, where data from a related, well-studied species can contribute to improved antigen identification by machine learning models.

## Methods

### Data collection

Protein sequences from the *P. vivax* P01 and *P. falciparum* 3D7 strains were extracted from PlasmoDB^54^ release 45 (2019-09-05) and release 43 (2019-04-25), respectively. Proteins with stop codons or “X” symbols in their sequences or those derived from pseudogenes were filtered out. Selenocysteines were replaced with cysteines to support downstream bioinformatic analyses. The resulting 6491 *P. vivax* and 5393 *P. falciparum* proteins were subsequently analyzed. The protein variables, to be used as input for the machine learning model, were gathered either from public databases or analyzed using various bioinformatics programs, as detailed below. The resulting 272 variables were categorized into four groups: genomic, immunological, proteomic, and structural. These variables were stored in an in-house database^21^ (MariaDB version 10.3.22, https://mariadb.com/) for facile data manipulation.

The genomic variables included data related to single nucleotide polymorphisms (SNPs), which were analyzed using whole genome sequencing and were directly downloaded from the genetic variation section in PlasmoDB^54^ (235 for *P. vivax* and 365 for *P. falciparum*). Various measurements of SNPs were considered, such as the total number of SNPs, the numbers of non-synonymous, synonymous, nonsense, and non-coding SNPs, the ratio of non-synonymous to synonymous SNPs, and the number of SNPs per kilobase of the coding sequence. Immunological variables comprised predictions of various epitopes such as T-cell epitopes^55^, B-cell epitopes^56–58^, cytotoxic T-cell epitopes^59^, chemokine inducer epitopes^60,61^, and transporter associated with antigen processing (TAP) binding peptides^62^. Additionally, they contained major histocompatibility complex (MHC) class I epitopes^63^, MHC class II epitopes^64^, as well as assessments of antigenicity^65^ and immunogenicity^66^. The epitope-related data were summarized based on numbers, maximum, mean, and minimum scores of epitopes within the protein sequence. Proteomic variables consisted of predictions regarding subcellular localization^67^, malarial adhesins/adhesin-like proteins^68^, and a set of physicochemical properties^69–71^, such as length, weight, isoelectric point, percentage of hydrophobic amino acids. Further, predictions of glycosylphosphatidylinositol (GPI)-anchored proteins^72^, signal cleavage^43^, protein solubility^73^, N-linked or O-linked glycosylation sites^74^, and similarity to human proteins^75^ were also included. Structural variables contained transmembrane helix predictions^76^, sequence complexity^77^, and predictions of beta turn^78^, surface accessibility^79^, and flexibility^80^. Relevant variables were subsequently integrated to construct additional variables. For instance, epitope predictions were combined with transmembrane predictions to derive variables that represent epitopes in outer, inner, or transmembrane regions. Refer to Supplementary Data File 1 for further detailed information about these variables.

### Known antigen labeling

Known antigens were collected from both literature and the Immune Epitope Database (IEDB). The web-based tool Covidence (www.covidence.org) was used to select relevant papers or documents using the search terms “malaria vaccine”, “malaria vaccine candidate”, “malaria vaccine antigen”, and “malaria vaccine protein”. To ensure the quality of known antigen labeling, each of the resulting papers and documents was manually reviewed to select the malaria vaccine candidates for inclusion as known antigens in this study. For IEDB-based known antigen labeling, data were retrieved from PlasmoDB under the immunology section. Known antigens were selected if the PlasmoDB proteins exhibited a similarity score of 97% or higher with GenBank proteins in the IEDB, and if all corresponding epitopes exactly matched the PlasmoDB protein sequence. For *P. falciparum*, 177 known antigens were selected from the literature and 373 from IEDB. For *P. vivax*, 24 known antigens were chosen from literature and 20 from IEDB. The known antigens for *P. falciparum* were determined by the intersection of the two sources, containing 52 known antigens. To get a comparable number of known antigens for *P. vivax*, the union of the two sources was computed, resulting in 38 known antigens.

### Machine learning data assembly and data combinations

Protein variable data for *P. vivax* were retrieved from our in-house database, with antigen labels appended as an additional column. In this label column, known antigens were assigned a value of one, and the remaining proteins were marked with a value of zero (Supplementary Data File 2). The data from *P. vivax* and *P. falciparum* were merged to train a combined model (Supplementary Data File 4). Additionally, two variants of combined data were created by incorporating subsets of data from each species. First, data containing both autologous proteins and heterologous positives were generated either by combining *P. falciparum* known antigens with the *P. vivax* data set or vice versa. Second, data comprising autologous proteins and heterologous unlabeled proteins were obtained either by adding *P. falciparum* unlabeled proteins to *P. vivax* data set or vice versa. Refer to Table 2 for a detailed overview of models and their corresponding input data.

### Positive-unlabeled random forest training

The positive-unlabeled random forest (PURF) algorithm^22^ has been optimized and tailored specifically to tackle the antigen identification problem^21^. PURF has the advantages inherited from conventional random forest, such as resilience to errors, insensitive to outliers, and high predictive power. Given the scarcity of known antigens, we enforced learning by implementing a variable space weighting process. In this process, known antigens were weighted based on the variable space, thereby enhancing their representations. Specifically, the center point of the known antigens in the variable space was computed and the Euclidean distances from each known antigen to this center point were scaled into integer values ranging between 1 and 10. Through this approach, known antigens were duplicated based on the integer weights, resulting in a total of 83 known antigens labeled as positive in the *P. vivax* data set. Regarding the combined data set, which includes known antigens from both *P. vivax* and *P. falciparum*, the total number of known antigens was 181.

PURF models, each composed of 100,000 trees, were independently trained on the *P. vivax* and combined data sets, including the variants of the combined data. The ensemble constituent filtering procedure was subsequently applied to the trained PURF models to further prioritize the top-scored unlabeled proteins, guided by the selected reference antigens. The reference antigens for *P. vivax* included the circumsporozoite protein (CSP, PVP01_0835600.1-p1), the Duffy binding protein (DBP, PVP01_0623800.1-p1), and the merozoite surface protein-1 (MSP-1, PVP01_0728900.1-p1). For *P. falciparum*, the reference antigens were the circumsporozoite protein (CSP, PF3D7_0304600.1-p1), the merozoite surface protein-5 (MSP-5, PF3D7_0206900.1-p1), the transmission-blocking target antigen s230 (P230, PF3D7_0209000.1-p1), and the reticulocyte binding homolog 5 (RH5, PF3D7_0424100.1-p1). Briefly, any trees that either lacked all reference antigens in the out-of-bag set or incorrectly predicted the reference antigens in the out-of-bag set were discarded. For the *P. vivax* model, the resulting number of trees was 93,102, and for the combined model, 86,254 trees remained after the filtering process. See Supplementary Data Files 3, 5 for the out-of-bag probability scores (proportion of votes) of the *P. vivax* and the combined models.

### Positive-unlabeled random forest evaluation

To optimize the *P. vivax* and the combined models, a retraining process on the positive level hyper-parameter was conducted. The positive level describes the prior proportion of potential antigens in the proteome, assuming the positives were labeled randomly. The positive level was used to estimate the number of positives in the children nodes based on the modified Gini splitting criterion when constructing a tree model^21^. The process involved training the models across a spectrum of positive level values, ranging from 0.1 and incremented by 0.1 until 0.9. The models were then evaluated with two defined criteria^21^. The first criterion relied on the probability score distribution generated from the PURF model. A two-component Gaussian mixture model was utilized to estimate the putative true and false positive rates from the distribution, and the area under the receiver operating characteristic (AUROC) curve was subsequently computed. The second criterion examined the percentile ranks of the known antigens within the entire proteome based on the probability scores, and the explicit positive recall (EPR)^24,28^ was calculated as well. The percentile ranks were also visualized with respect to the antigens ranked by probability scores, and the area under the curve (AUC) was computed. Based on the two criteria, a positive level of 0.5 was selected for both the *P. vivax* and the combined models. Although the precision and thus the *F*_*1*_ score cannot be computed directly for a positive-unlabeled dataset, an alternative metric, similar to the *F*_*1*_ score, can be estimated using the following formula^23^:

$$\begin{array}{c}\frac{{precision}\,\cdot \,{recall}}{\Pr \left(y=1\right)}\\ \begin{array}{c}\,=\,\frac{{precision}\,\cdot \,{{recall}}^{2}}{{recall}\,\cdot \,\Pr \left(y\,=\,1\right)}\\ \begin{array}{c}\,=\,\frac{\Pr \left(y\,=\,1\left|\hat{y}\,=\,1\right.\right)\,\cdot \,{{recall}}^{2}}{\Pr \left(\hat{y}\,=\,1,y\,=\,1\right)}\\ =\frac{{{recall}}^{2}}{\Pr \left(\hat{y}\,=\,1\right)}\end{array}\end{array}\end{array}$$

### Adversarial control analysis

To assess and validate the robustness of the models, adversarial controls^81^ were generated by changing the positive label to unlabeled for each known antigen in turn, excluding the reference antigens. In each iteration, after assigning a zero value to a known antigen, variable space weighting was applied to the altered data set. Subsequently, a model was trained, and the trees in the model were filtered using the ensemble constituent filtering procedure. Out-of-bag probability scores were then computed for the remaining known antigens that did not have their labels removed. As the reference antigens were excluded from the analysis, there were 35 adversarial control models generated for the *P. vivax* data set, and 83 for the combined data set. The resulting data were further analyzed in two ways. First, the scores of known antigens in each adversarial control had the baseline scores derived from the original models subtracted from them, following which the mean difference was calculated. The mean differences in scores from all adversarial controls were then compared across the *P. vivax*, *P. falciparum*, and the combined data sets. Second, for each adversarial control model, the accuracy of the remaining known antigens was computed using a probability score threshold of 0.5. The results of all adversarial control models were summarized as a mean and standard deviation, and compared across the *P. vivax*, *P falciparum*, and the combined data sets. To further investigate the two modes observed in the distribution of differences in scores, a two-sided Fisher’s exact test was performed using the function *fisher.test* in R stats, version 4.2.3. This test was utilized to compare the two modes with either species types or known antigen source, where the odds ratio and *p*-value were subsequently calculated.

### Comparison of models trained with different data combinations

As described in the above section in Methods, variants of combined data were generated by integrating portions of data from *P. vivax* and *P. falciparum*. Multiple PURF models were compared to understand the impact of autologous and heterologous data. For each species, these models included the single species model (which can be viewed as either an autologous or heterologous model, depending on the species for which the prediction score were generated), the combined model, the autologous with heterologous positives model, and the autologous with unlabeled model. Models were then compared based on scores of known antigens and predictions of unlabeled proteins. For each species, the score distribution for each model was visualized by plotting the densities and boxplots of the known antigens, along with predicted antigens and non-antigens, as shown in Fig. 3. Known antigens were further quantified using EPR and percentile ranks, where the AUC values were computed. Subsequently, the predictions for unlabeled proteins were compared by examining the association between antigen predictions and species types using a *χ*^2^ test, utilizing the function *chisq.test* in R stats, version 4.2.3. The strength of the association was further analyzed using Cramér’s V, using the function *cramerV* in the R package rcompanion, version 2.4.30^82^. The 95% confidence interval of Cramér’s V was calculated using a bootstrap approach with 1,000 replications. Additionally, the mean tree depths across all trees in each type of model were calculated and compared to the proportion of positives in the training data set. A linear model was fitted using the *lm* function in R stats, version 4.2.3. The dependent variable *mean tree depth* was log_2_-transformed, and the *logit* function was applied to the independent variable *proportion of positives*. The regression function was log(*y*) = 6.8 + 0.87 · logit(*x*), and the adjusted *R*^2^ was 0.7. The *p*-value derived from the *F*-test was 0.01.

### Model interpretation of the combined model

A proximity matrix quantifying closeness between proteins in the model was computed. Specifically, for any pair of proteins in the training data set, the proximity value was calculated by counting the times the pair was in the out-of-bag set and ended in the same leaf across all trees. The proximity value was then normalized by dividing the value by the number of times the pair of proteins was in the out-of-bag set. The proximity value ranges from zero to one, which was then transformed into a Euclidean distance by subtracting its value from one, resulting in a dissimilarity matrix. To further reduce the dimensions of the dissimilarity matrix, multidimensional scaling, also known as principal coordinates analysis, was applied using the *cmdscale* function in R stats, version 4.2.3. For enhanced visualization, a two-dimensional representation was constructed by employing the uniform manifold approximation and projection (UMAP)^83^ method with two components.

### Clustering and amino acid composition analyses of model predictions

To further explore the separation of predicted antigens observed in the aforementioned visualization of the model predictions based on the dissimilarity matrix, the Ward’s hierarchical agglomerative clustering method^84^ was utilized to analyze the data, using the R stats function *hclust*, along with the “ward.D2” implementation^85^. A dendrogram was generated through the hierarchical clustering analysis, and an iterative process was initiated to segregate predicted antigens into groups. In each iteration, the predicted antigens were split into two groups according to the dendrogram. To assess the association between the groups and the species types, a *χ*^2^ test and Cramér’s V were computed. A sub-dendrogram was then created from one of the two groups having the higher average probability score, and the iterative process continued. A total of four iterations were generated, yielding *χ*^2^ *p*-values of 1.11 × 10^−10^, 6.50 × 10^−46^, <2.23 × 10^−308^, and 0.33. The Cramér’s V values, along with the 95% confidence interval (CI) values, were 0.09 (95% CI: 0.06, 0.11), 0.24 (95% CI: 0.21, 0.27), 0.94 (95% CI: 0.93, 0.95), and 0.02 (95% CI: 0.00, 0.08). The resulting groups were further visualized on the UMAP representation of the dimension-reduced dissimilarity matrix, as detailed previously in the model interpretation section. Finally, to evaluate the association between amino acid composition and species types, an association analysis was conducted to analyze the two variables in three groups: the whole proteome, the predicted antigens, and the predicted non-antigens. For the whole proteome group, the frequencies of the 20 amino acids were independently computed for the proteomes of *P. vivax* and *P. falciparum*. For the predicted antigen and non-antigen groups, the amino acid frequencies of the two species were computed for each group as well. In each comparison group, the association between the amino acid frequencies and the species types was evaluated using a *χ*^2^ test and Cramér’s V. Additionally, 84 orthologs of the 52 *P. falciparum* known antigens were collected from OrtholoMCL-DB^86^, release 6.20 (2024-02-21), which detects orthologs through a genome-scale algorithm computed on protein sequences. Another set of 84 top *P. vivax* candidates were extracted based on the combined model predictions. The prediction scores of the *P. vivax* and combined models for the orthologs and top candidates were computed, and a two-sided Mann–Whitney test was performed for each of the ortholog and candidate groups. The Benjamini–Hochberg^87^ method was applied to adjust the *p*-values.

### Variable importance analysis

To understand the important variables in model predictions, a permutation-based variable importance analysis was performed on the trained PURF models using the method proposed by Breiman^29^. To explore patterns in variable values, the values were first normalized to range between 0 and 1 for each variable. A set of predicted non-antigens with the same size as the known antigens in the training data set was then randomly selected. Next, to compare the variable values between the known antigens and the predicted non-antigens that were randomly selected, a two-sided Mann–Whitney test was performed. The Benjamini–Hochberg^87^ method was applied to adjust the *p*-values for comparisons of all 272 variables. Additionally, a permutation-based group variable importance analysis was conducted for the four variable groups: genomic, immunological, proteomic, and structural. The process was the same as described earlier except that variables within the same group were permuted together to compute their collective impact on prediction accuracy. Finally, top 10 important variables in the *P. vivax*, *P. falciparum*, and combined models were compared using a Venn diagram. To understand how the top 10 important variables of the combined model influence the prediction accuracy of the known antigens in the single-species models, importance values of this identical variable set were compared across all three models.

### Clustering of top candidate antigens

Top candidate antigens were selected based on a probability score threshold, where half of the known antigens were scored above this threshold, resulting in 190 potential vaccine antigen candidates. A dissimilarity matrix of these top candidate antigens, computed from the tree-based structures in the model, was then analyzed using Ward’s hierarchical agglomerative clustering method^85^, as previously described. The number of clustering groups was determined using Gap statistic with the Tibshirani criterion^88,89^, Silhouette^30^, and Elbow (or total within sum of square) methods. These methods identified 1, 2, and 3 groups, respectively. As a result, top candidate antigens were clustered into either two or three groups. The groups were then visualized on a two-dimensional UMAP representation. For the clustering with two groups based on the Silhouette method, group 1 contained 35 *P. vivax* and 0 *P. falciparum* candidate antigens, while group 2 had 10 *P. vivax* and 145 *P. falciparum* candidates. For the clustering with three groups based on the Elbow method, group 1 contained 35 *P. vivax* and 0 *P. falciparum* candidates, group 2 comprised 9 *P. vivax* and 107 *P. falciparum* candidates, and group 3 consisted of 1 *P. vivax* and 38 *P. falciparum* candidates. Orthologs were identified through searching in PlasmoDB, which was based on the data set generated using the OrthoMCL algorithm^86,90^. A summary table was subsequently generated to display information of the top candidate antigens, which include the associated clustering groups, probability scores, gene products (retrieved from PlasmoDB^54^, release 62), the closest known antigens and their source, as well as the Euclidean distance (ranging from 0 to 1) to the closest known antigen. Further details can be found in Supplementary Data File 6.

### Gene ontology enrichment analysis

To better understand gene ontology (GO) terms associated with the three groups of candidate antigens, an enrichment analysis was conducted using the Python package *GOATOOLS*^91^. The gene ontology terms for *P. vivax* and *P. falciparum* were downloaded directly from PlasmoDB^54^, release 62 (2023-03-09). The file containing the directed acyclic graph of gene ontology was retrieved from the Gene Ontology website (http://geneontology.org/docs/download-ontology/)^92,93^. For more conservative results, the argument *propagate_counts* in the function *GOEnrichmentStudyNS* was set as false. GO enrichment analysis was performed with the background proteomes from both *P. vivax* and *P. falciparum* species. The *p*-values from the multiple Fisher’s exact tests were adjusted using the Benjamini–Hochberg^87^ method. Enriched GO terms, identified based on the significant cut-off of 0.5, were categorized into biological process, cellular component, and molecular function, and were further visualized for each of the three candidate groups.

## Supplementary information

Supplementary information

Supplementary Data 1

Supplementary Data 2

Supplementary Data 3

Supplementary Data 4

Supplementary Data 5

Supplementary Data 6

## Data Availability

All key resources, including the database file, source data for figures and tables, as well as the research notebook, have been deposited in the Digital Repository at the University of Maryland (DRUM), 10.13016/dspace/vijt-jshg. The interactive candidate antigen table can be access online at https://mrp-bioinformatics.github.io/malaria_antigen_candidates/.

## Code availability

The code for executing the machine learning algorithms, data analyses, and figure generation, is contained within a research notebook, and has been archived in DRUM, 10.13016/dspace/vijt-jshg, and GitHub, https://rtchou.github.io/malaria-antigen-identification-pv/.

## References

[1] WHO. World malaria report 2022. *World Health Organization, Geneva* (2022).

[2] Balikagala, B. et al. Evidence of artemisinin-resistant malaria in Africa. *N. Engl. J. Med.* **385**, 1163–1171 (2021). 34551228 10.1056/NEJMoa2101746

[3] Moyes, C. L. et al. Evaluating insecticide resistance across African districts to aid malaria control decisions. *Proc. Natl Acad. Sci. USA* **117**, 22042–22050 (2020). 32843339 10.1073/pnas.2006781117 PMC7486715

[4] Nass, J. & Efferth, T. Development of artemisinin resistance in malaria therapy. *Pharm. Res.* **146**, 104275 (2019). 10.1016/j.phrs.2019.104275 31100335

[5] Plowe, C. V., Alonso, P. & Hoffman, S. L. The potential role of vaccines in the elimination of *falciparum* malaria and the eventual eradication of malaria. *J. Infect. Dis.* **200**, 1646–1649 (2009). 19877844 10.1086/646613

[6] Henderson, D. A. Lessons from the eradication campaigns. *Vaccine* **17**, S53–S55 (1999). 10559535 10.1016/s0264-410x(99)00293-5

[7] Mueller, I., Shakri, A. R. & Chitnis, C. E. Development of vaccines for *Plasmodium vivax* malaria. *Vaccine* **33**, 7489–7495 (2015). 26428453 10.1016/j.vaccine.2015.09.060

[8] Beeson, J. G. et al. Challenges and strategies for developing efficacious and long-lasting malaria vaccines. *Sci. Transl. Med.* **11**, 1–17 (2019). 10.1126/scitranslmed.aau1458 30626712

[9] Galinski, M. R. & Barnwell, J. W. *Plasmodium vivax*: who cares? *Malar. J.* **7**, S9 (2008). 19091043 10.1186/1475-2875-7-S1-S9 PMC2604873

[10] Neafsey, D. E. et al. The malaria parasite *Plasmodium vivax* exhibits greater genetic diversity than *Plasmodium falciparum*. *Nat. Genet* **44**, 1046–1050 (2012). 22863733 10.1038/ng.2373 PMC3432710

[11] Neafsey, D. E. et al. Genetic diversity and protective efficacy of the RTS,S/AS01 malaria vaccine. *N. Engl. J. Med.* **373**, 2025–2037 (2015). 26488565 10.1056/NEJMoa1505819 PMC4762279

[12] Takala, S. L. & Plowe, C. V. Genetic diversity and malaria vaccine design, testing and efficacy: preventing and overcoming ‘vaccine resistant malaria’. *Parasite Immunol.* **31**, 560–573 (2009). 19691559 10.1111/j.1365-3024.2009.01138.x PMC2730200

[13] Rappuoli, R. & Covacci, A. Reverse vaccinology and genomics. *Science* **302**, 602 (2003). 14576423 10.1126/science.1092329

[14] Moxon, R., Reche, P. A. & Rappuoli, R. Editorial: reverse vaccinology. *Front Immunol.* **10**, 2776 (2019). 31849959 10.3389/fimmu.2019.02776 PMC6901788

[15] Pizza, M. et al. Identification of vaccine candidates against serogroup B meningococcus by whole-genome sequencing. *Science* **287**, 1816–1820 (2000). 10710308 10.1126/science.287.5459.1816

[16] Tettelin, H. et al. Complete genome sequence of *Neisseria meningitidis* serogroup B strain MC58. *Science* **287**, 1809–1815 (2000). 10710307 10.1126/science.287.5459.1809

[17] Rappuoli, R. Reverse vaccinology, a genome-based approach to vaccine development. *Vaccine* **19**, 2688–2691 (2001). 11257410 10.1016/s0264-410x(00)00554-5

[18] Sette, A. & Rappuoli, R. Reverse vaccinology: developing vaccines in the era of genomics. *Immunity* **33**, 530–541 (2010). 21029963 10.1016/j.immuni.2010.09.017 PMC3320742

[19] Singh, S. P., Srivastava, D. & Mishra, B. N. Genome-wide identification of novel vaccine candidates for *Plasmodium falciparum* malaria using integrative bioinformatics approaches. *3 Biotech* **7**, 318 (2017). 28955615 10.1007/s13205-017-0947-7 PMC5602874

[20] Pritam, M., Singh, G., Swaroop, S., Singh, A. K. & Singh, S. P. Exploitation of reverse vaccinology and immunoinformatics as promising platform for genome-wide screening of new effective vaccine candidates against *Plasmodium falciparum*. *BMC Bioinforma.* **19**, 468 (2019). 10.1186/s12859-018-2482-x PMC7394322 30717656

[21] Chou, R. T. et al. Positive-unlabeled learning identifies vaccine candidate antigens in the malaria parasite *Plasmodium falciparum*. *npj Syst. Biol. Appl* **10**, 44 (2024). 38678051 10.1038/s41540-024-00365-1 PMC11055854

[22] Li, C. & Hua, X.-L. Towards positive unlabeled learning for parallel data mining: a random forest framework. *Int Conf Adv Comput Appl*, 573-587 (2014).

[23] Bekker, J. & Davis, J. Learning from positive and unlabeled data: a survey. *Mach. Learn* **109**, 719–760 (2020).

[24] Li, F. et al. Positive-unlabeled learning in bioinformatics and computational biology: a brief review. *Brief. Bioinform* **23**, 1–13 (2022). 10.1093/bib/bbab461 34729589

[25] Xu, S., Kelkar, N. S. & Ackerman, M. E. Positive-unlabeled learning to infer pretection status and identify correlates in vaccine efficacy field trials. *iScience* **27**, 1–16 (2024). 10.1016/j.isci.2024.109086 PMC11409573 39295637

[26] Kelkar, N. S., Morrison, K. S. & Ackerman, M. E. Foundations for improved vaccine correlate of risk analysis using positive-unlabeled learning. *Hum. Vaccin Immunother.* **19**, 1–11 (2023). 10.1080/21645515.2023.2204020 PMC10294723 37133899

[27] Vita, R. et al. The immune epitope database (IEDB) 3.0. *Nucleic Acids Res.* **43**, D405–D412 (2015). 25300482 10.1093/nar/gku938 PMC4384014

[28] Cheng, Z., Zhou, S. & Guan, J. Computationally predicting protein-RNA interactions using only positive and unlabeled examples. *J. Bioinform Comput Biol.* **13**, 1541005 (2015). 25790785 10.1142/S021972001541005X

[29] Breiman, L. Random forests. *Mach. Learn* **45**, 5–32 (2001).

[30] Rousseeuw, P. J. Silhouettes: a graphical aid to the interpretation and validation of cluster analysis. *J. Comput Appl Math.* **20**, 53–65 (1987).

[31] Roh, Y., Heo, G. & Whang, S. E. A survey on data collection for machine learning: a big data - AI integration perspective. *IEEE Trans. Knowl. Data Eng.* **33**, 1328–1347 (2019).

[32] Bjorkman, A., Benn, C. S., Aaby, P. & Schapira, A. RTS,S/AS01 malaria vaccine-proven safe and effective? *Lancet Infect. Dis.* **23**, e318–e322 (2023). 37086747 10.1016/S1473-3099(23)00126-3

[33] Datoo, M. S. et al. Efficacy and immunogenicity of R21/Matrix-M vaccine against clinical malaria after 2 years’ follow-up in children in Burkina Faso: a phase 1/2b randomised controlled trial. *Lancet Infect. Dis.* **22**, 1728–1736 (2022). 36087586 10.1016/S1473-3099(22)00442-X

[34] da Veiga, G. T. S., Moriggi, M. R., Vettorazzi, J. F., Muller-Santos, M. & Albrecht, L. *Plasmodium vivax* vaccine: what is the best way to go? *Front Immunol.* **13**, 910236 (2022). 36726991 10.3389/fimmu.2022.910236 PMC9885200

[35] Bermudez, M., Moreno-Perez, D. A., Arevalo-Pinzon, G., Curtidor, H. & Patarroyo, M. A. *Plasmodium vivax* in vitro continuous culture: the spoke in the wheel. *Malar. J.* **17**, 301 (2018). 30126427 10.1186/s12936-018-2456-5 PMC6102941

[36] Aguttu, C., Okech, B. A., Mukisa, A. & Lubega, G. W. Screening and characterization of hypothetical proteins of *Plasmodium falciparum* as novel vaccine candidates in the fight against malaria using reverse vaccinology. *J. Genet Eng. Biotechnol.* **19**, 103 (2021). 34269931 10.1186/s43141-021-00199-y PMC8283385

[37] Goodswen, S. J., Kennedy, P. J. & Ellis, J. T. A novel strategy for classifying the output from an in silico vaccine discovery pipeline for eukaryotic pathogens using machine learning algorithms. *BMC Bioinforma.* **14**, 315 (2013). 10.1186/1471-2105-14-315 PMC3826511 24180526

[38] Rodrigues-da-Silva, R. N. et al. In silico identification and validation of a linear and naturally immunogenic B-cell epitope of the *Plasmodium vivax* malaria vaccine candidate merozoite surface protein-9. *PLoS One* **11**, e0146951 (2016). 26788998 10.1371/journal.pone.0146951 PMC4720479

[39] Hostetler, J. B. et al. A library of *Plasmodium vivax* recombinant merozoite proteins reveals new vaccine candidates and protein-protein interactions. *PLoS Negl. Trop. Dis.* **9**, e0004264 (2015). 26701602 10.1371/journal.pntd.0004264 PMC4689532

[40] Siegel, S. V. et al. Analysis of *Plasmodium vivax* schizont transcriptomes from field isolates reveals heterogeneity of expression of genes involved in host-parasite interactions. *Sci. Rep.* **10**, 16667 (2020). 33028892 10.1038/s41598-020-73562-7 PMC7541449

[41] Kundu, P. et al. The structure of a *Plasmodium vivax* Tryptophan Rich Antigen suggests a lipid binding function for a pan-*Plasmodium* multi-gene family. *Nat. Commun.* **14**, 1–17 (2023). 37709739 10.1038/s41467-023-40885-8 PMC10502043

[42] Goodswen, S. J., Kennedy, P. J. & Ellis, J. T. A guide to current methodology and usage of reverse vaccinology towards in silico vaccine discovery. *FEMS Microbiol Rev.* **47**, 1–22 (2023). 10.1093/femsre/fuad004 36806618

[43] Almagro Armenteros, J. J. et al. SignalP 5.0 improves signal peptide predictions using deep neural networks. *Nat. Biotechnol.* **37**, 420–423 (2019). 30778233 10.1038/s41587-019-0036-z

[44] Hamilton, W. L. et al. Extreme mutation bias and high AT content in *Plasmodium falciparum*. *Nucleic Acids Res.* **45**, 1889–1901 (2017). 27994033 10.1093/nar/gkw1259 PMC5389722

[45] Rappuoli, R. Reverse vaccinology. *Curr. Opin. Microbiol* **3**, 445–450 (2000). 11050440 10.1016/s1369-5274(00)00119-3

[46] Hayashida, K. et al. Direct detection of *falciparum* and non-*falciparum* malaria DNA from a drop of blood with high sensitivity by the dried-LAMP system. *Parasit. Vectors* **10**, 26 (2017). 28086864 10.1186/s13071-016-1949-8 PMC5237333

[47] Woldearegai, T. G. et al. Characterization of *Plasmodium* infections among inhabitants of rural areas in Gabon. *Sci. Rep.* **9**, 9784 (2019). 31278305 10.1038/s41598-019-46194-9 PMC6611864

[48] Taylor, S. M. et al. Molecular malaria epidemiology: mapping and burden estimates for the Democratic Republic of the Congo, 2007. *PLoS One* **6**, e16420 (2011). 21305011 10.1371/journal.pone.0016420 PMC3031549

[49] Sitali, L. et al. Distribution of *Plasmodium* species and assessment of performance of diagnostic tools used during a malaria survey in Southern and Western Provinces of Zambia. *Malar. J.* **18**, 130 (2019). 30971231 10.1186/s12936-019-2766-2 PMC6458729

[50] White, N. J. *Plasmodium knowlesi*: the fifth human malaria parasite. *Clin. Infect. Dis.* **46**, 172–173 (2008). 18171246 10.1086/524889

[51] Chin, A. Z. et al. Malaria elimination in Malaysia and the rising threat of *Plasmodium knowlesi*. *J. Physiol. Anthropol.* **39**, 36 (2020). 33228775 10.1186/s40101-020-00247-5 PMC7686722

[52] Cooper, D. J. et al. *Plasmodium knowlesi* malaria in Sabah, Malaysia, 2015-2017: ongoing increase in incidence despite near-elimination of the human-only *Plasmodium* species. *Clin. Infect. Dis.* **70**, 361–367 (2020). 30889244 10.1093/cid/ciz237 PMC7768742

[53] Pongvongsa, T. et al. Human infection with *Plasmodium knowlesi* on the Laos-Vietnam border. *Trop. Med Health* **46**, 33 (2018). 30250398 10.1186/s41182-018-0116-7 PMC6145087

[54] Aurrecoechea, C. et al. PlasmoDB: a functional genomic database for malaria parasites. *Nucleic Acids Res.* **37**, D539–D543 (2009). 18957442 10.1093/nar/gkn814 PMC2686598

[55] Oyarzun, P., Ellis, J. J., Boden, M. & Kobe, B. PREDIVAC: CD4+ T-cell epitope prediction for vaccine design that covers 95% of HLA class II DR protein diversity. *BMC Bioinforma.* **14**, 52 (2013). 10.1186/1471-2105-14-52 PMC3598884 23409948

[56] Jespersen, M. C., Peters, B., Nielsen, M. & Marcatili, P. BepiPred-2.0: improving sequence-based B-cell epitope prediction using conformational epitopes. *Nucleic Acids Res.* **45**, W24–W29 (2017). 28472356 10.1093/nar/gkx346 PMC5570230

[57] Larsen, J. E., Lund, O. & Nielsen, M. Improved method for predicting linear B-cell epitopes. *Immunome Res.* **2**, 2 (2006). 16635264 10.1186/1745-7580-2-2 PMC1479323

[58] Saha, S. & Raghava, G. P. Prediction of continuous B-cell epitopes in an antigen using recurrent neural network. *Proteins* **65**, 40–48 (2006). 16894596 10.1002/prot.21078

[59] Bhasin, M. & Raghava, G. P. Prediction of CTL epitopes using QM, SVM and ANN techniques. *Vaccine* **22**, 3195–3204 (2004). 15297074 10.1016/j.vaccine.2004.02.005

[60] Nagpal, G. et al. Computer-aided designing of immunosuppressive peptides based on IL-10 inducing potential. *Sci. Rep.* **7**, 42851 (2017). 28211521 10.1038/srep42851 PMC5314457

[61] Dhanda, S. K., Vir, P. & Raghava, G. P. Designing of interferon-gamma inducing MHC class-II binders. *Biol. Direct* **8**, 30 (2013). 24304645 10.1186/1745-6150-8-30 PMC4235049

[62] Bhasin, M. & Raghava, G. P. Analysis and prediction of affinity of TAP binding peptides using cascade SVM. *Protein Sci.* **13**, 596–607 (2004). 14978300 10.1110/ps.03373104 PMC2286721

[63] Nielsen, M. et al. Reliable prediction of T-cell epitopes using neural networks with novel sequence representations. *Protein Sci.* **12**, 1007–1017 (2003). 12717023 10.1110/ps.0239403 PMC2323871

[64] Bui, H. H. et al. Automated generation and evaluation of specific MHC binding predictive tools: ARB matrix applications. *Immunogenetics* **57**, 304–314 (2005). 15868141 10.1007/s00251-005-0798-y

[65] Kolaskar, A. S. & Tongaonkar, P. C. A semi-empirical method for prediction of antigenic determinants on protein antigens. *FEBS Lett.* **276**, 172–174 (1990). 1702393 10.1016/0014-5793(90)80535-q

[66] Calis, J. J. et al. Properties of MHC class I presented peptides that enhance immunogenicity. *PLoS Comput Biol.* **9**, e1003266 (2013). 24204222 10.1371/journal.pcbi.1003266 PMC3808449

[67] Yu, C. S., Chen, Y. C., Lu, C. H. & Hwang, J. K. Prediction of protein subcellular localization. *Proteins* **64**, 643–651 (2006). 16752418 10.1002/prot.21018

[68] Ansari, F. A., Kumar, N., Bala Subramanyam, M., Gnanamani, M. & Ramachandran, S. MAAP: malarial adhesins and adhesin-like proteins predictor. *Proteins* **70**, 659–666 (2008). 17879344 10.1002/prot.21568

[69] Osorio, D. & Rondón-Villarrea, P. Peptides: a package for data mining of antimicrobial peptides. *R. J.* **7**, 4–14 (2015).

[70] Xiao, N., Cao, D. S., Zhu, M. F. & Xu, Q. S. protr/ProtrWeb: R package and web server for generating various numerical representation schemes of protein sequences. *Bioinformatics* **31**, 1857–1859 (2015). 25619996 10.1093/bioinformatics/btv042

[71] Parker, J. M., Guo, D. & Hodges, R. S. New hydrophilicity scale derived from high-performance liquid chromatography peptide retention data: correlation of predicted surface residues with antigenicity and X-ray-derived accessible sites. *Biochemistry* **25**, 5425–5432 (1986). 2430611 10.1021/bi00367a013

[72] Pierleoni, A., Martelli, P. L. & Casadio, R. PredGPI: a GPI-anchor predictor. *BMC Bioinforma.* **9**, 392 (2008). 10.1186/1471-2105-9-392 PMC2571997 18811934

[73] Hebditch, M. & Warwicker, J. Charge and hydrophobicity are key features in sequence-trained machine learning models for predicting the biophysical properties of clinical-stage antibodies. *PeerJ* **7**, e8199 (2019). 31976163 10.7717/peerj.8199 PMC6967001

[74] Chauhan, J. S., Rao, A. & Raghava, G. P. In silico platform for prediction of N-, O- and C-glycosites in eukaryotic protein sequences. *PLoS One* **8**, e67008 (2013). 23840574 10.1371/journal.pone.0067008 PMC3695939

[75] Camacho, C. et al. BLAST+: architecture and applications. *BMC Bioinforma.* **10**, 421 (2009). 10.1186/1471-2105-10-421 PMC2803857 20003500

[76] Sonnhammer, E. L., von Heijne, G. & Krogh, A. A hidden Markov model for predicting transmembrane helices in protein sequences. *Proc. Int Conf. Intell. Syst. Mol. Biol.* **6**, 175–182 (1998). 9783223

[77] Wootton, J. C. & Federhen, S. Analysis of compositionally biased regions in sequence databases. *Methods Enzymol.* **266**, 554–571 (1996). 8743706 10.1016/s0076-6879(96)66035-2

[78] Chou, P. Y. & Fasman, G. D. Prediction of the secondary structure of proteins from their amino acid sequence. *Adv. Enzymol. Relat. Areas Mol. Biol.* **47**, 45–148 (1978). 364941 10.1002/9780470122921.ch2

[79] Emini, E. A., Hughes, J. V., Perlow, D. S. & Boger, J. Induction of hepatitis A virus-neutralizing antibody by a virus-specific synthetic peptide. *J. Virol.* **55**, 836–839 (1985). 2991600 10.1128/jvi.55.3.836-839.1985 PMC255070

[80] Karplus, P. & Schulz, G. Prediction of chain flexibility in proteins. *Naturwissenschaften* **72**, 212–213 (1985).

[81] Chuang, K. V. & Keiser, M. J. Adversarial controls for scientific machine learning. *ACS Chem. Biol.* **13**, 2819–2821 (2018). 30336670 10.1021/acschembio.8b00881

[82] Mangiafico, S. rcompanion: functions to support extension education program evaluation. *Rutgers Cooperative Extension* (2023).

[83] McInnes, L., Healy, J. & Melville, J. UMAP: uniform manifold approximation and projection for dimension reduction. *J Open Source Softw* (2018).

[84] Ward, J. H. Hierarchical grouping to optimize an objective function. *J Am Stat Assoc* (1963).

[85] Murtagh, F. & Legendre, P. Ward’s hierarchical agglomerative clustering method: which algorithms implement Ward’s criterion? *J. Classif.* **31**, 274–295 (2014).

[86] Chen, F., Mackey, A. J., Stoeckert, C. J. Jr. & Roos, D. S. OrthoMCL-DB: querying a comprehensive multi-species collection of ortholog groups. *Nucleic Acids Res.* **34**, D363–D368 (2006). 16381887 10.1093/nar/gkj123 PMC1347485

[87] Benjamini, Y. & Hochberg, Y. Controlling the false discovery rate: a practical and powerful approach to multiple testing. *J. R. Stat. Soc. B* **57**, 289–300 (1995).

[88] Tibshirani, R., Walther, G. & Hastie, T. Estimating the number of clusters in a dataset via the Gap statistic. *Technical Report, Stanford* (2000).

[89] Tibshirani, R., Walther, G. & Hastie, T. Estimating the number of data clusters via the Gap statistic. *J. R. Stat. Soc. Ser. B* **63**, 411–423 (2001).

[90] Li, L., Stoeckert, C. J. Jr. & Roos, D. S. OrthoMCL: identification of ortholog groups for eukaryotic genomes. *Genome Res.* **13**, 2178–2189 (2003). 12952885 10.1101/gr.1224503 PMC403725

[91] Klopfenstein, D. V. et al. GOATOOLS: a Python library for gene ontology analyses. *Sci. Rep.* **8**, 10872 (2018). 30022098 10.1038/s41598-018-28948-z PMC6052049

[92] Ashburner, M. et al. Gene ontology: tool for the unification of biolog. *y. Gene Ontol. Consort. Nat. Genet* **25**, 25–29 (2000). 10.1038/75556 PMC3037419 10802651

[93] Gene Ontology Consortium. The Gene Ontology resource: enriching a GOld mine. *Nucleic Acids Res.* **49**, D325–D334 (2021). 33290552 10.1093/nar/gkaa1113 PMC7779012

