# Supplementary material for: Plasmodium vivax antigen candidate prediction improves with the addition of Plasmodium falciparum data
Source: NPJ Syst Biol Appl. 2024 Nov 13;10:133. doi: 10.1038/s41540-024-00465-y (PMC11561111; doi:10.1038/s41540-024-00465-y)
Supplement: Supplementary file 1 — Supplementary information [file 41540_2024_465_MOESM1_ESM.pdf]

Plasmodium vivax antigen candidate prediction improves with the addition of *Plasmodium falciparum* data

Renee Ti Chou[1], Amed Ouattara[2], Shannon Takala-Harrison[2†], and Michael P. Cummings[1†]

[1]Center for Bioinformatics and Computational Biology, University of Maryland, College Park, College Park, MD, USA
[2]Center for Vaccine Development and Global Health, University of Maryland School of Medicine, Baltimore, MD, USA
[†]Corresponding authors. e-mail: mcummin1@umd.edu; stakala@som.umaryland.edu

1 Supplementary Notes

All supplementary materials, including the database file, raw data, and the research notebook, can be found in the Digital Repository at the University of Maryland (DRUM), `https://doi.org/10.13016/dspace/vijt-jshg`. The research notebook provides guidance on extracting protein variables, assembling machine learning input from the database, and code for conducting experimental analyses and creating plots. To open the research notebook, click on the HTML file `index.html` in the folder `main_notebook` to open it in a web browser. Alternatively, you can open the PDF version of the notebook `main_notebook.pdf` in the same folder. To execute the code in the research notebook, open the corresponding R Markdown (`.Rmd`) files in RStudio. The data generated from the notebook are stored in the following subfolders: `other_data` (structured data), `purf_models` (Python positive-unlabeled random forest models), and `rdata` (R objects).

2 Supplementary Figures

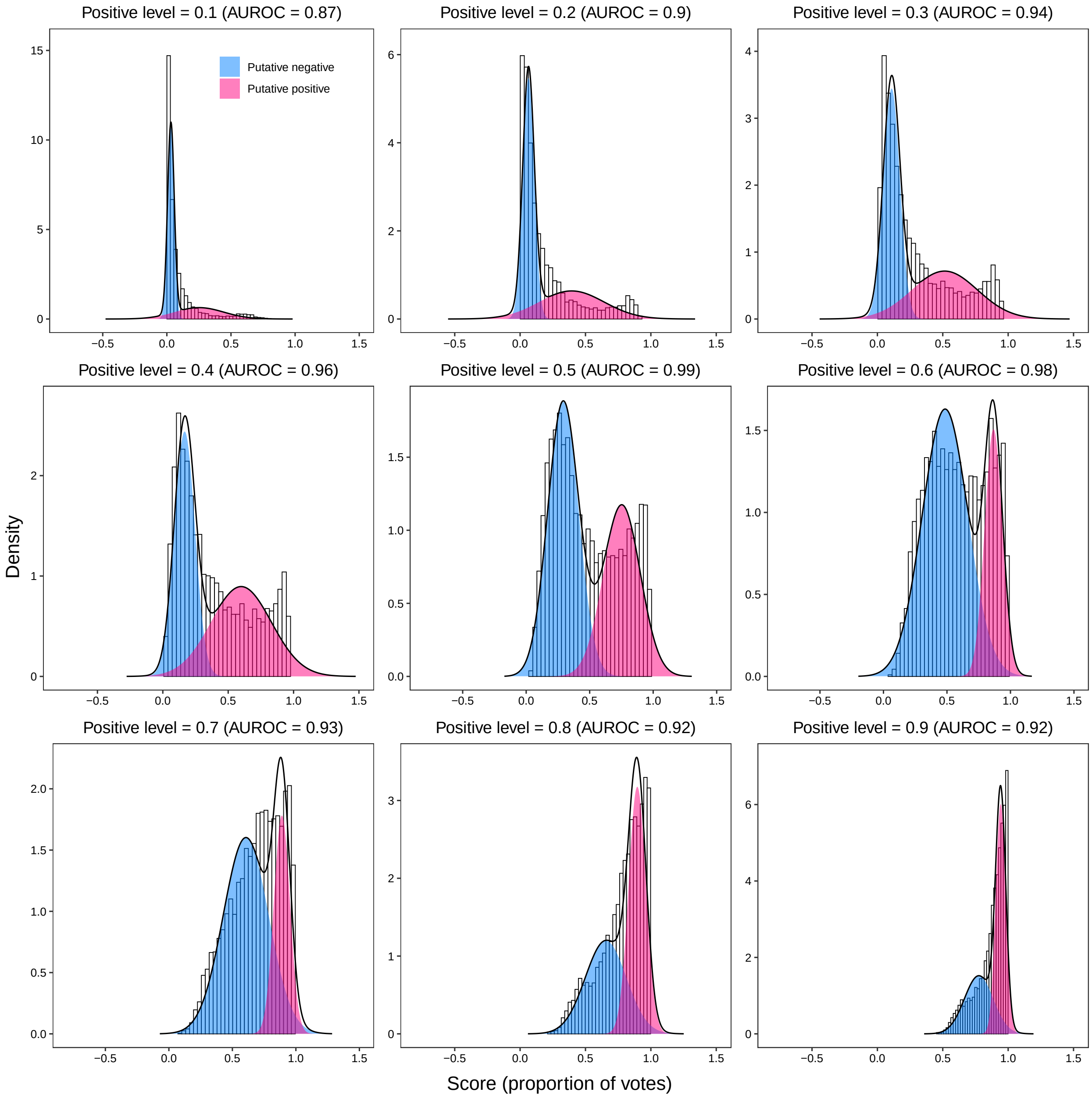

Supplementary Fig. 1 | Hyper-parameter tuning for PURF model trained on the *P. vivax* data set. Subplots showing probability score (proportion of votes) distributions of unlabeled proteins predicted by *P. vivax* models trained on different positive level (model hyper-parameter) settings ranging from 0.1 to 0.9. Magenta indicates putative positive distribution and blue represents putative negative distribution computed from a two-component Gaussian mixture model. Receiver operating characteristic (ROC) curves were computed based on the estimated distributions. The areas under the receiver operating characteristic curves (AUROC) are noted in the subplot titles.

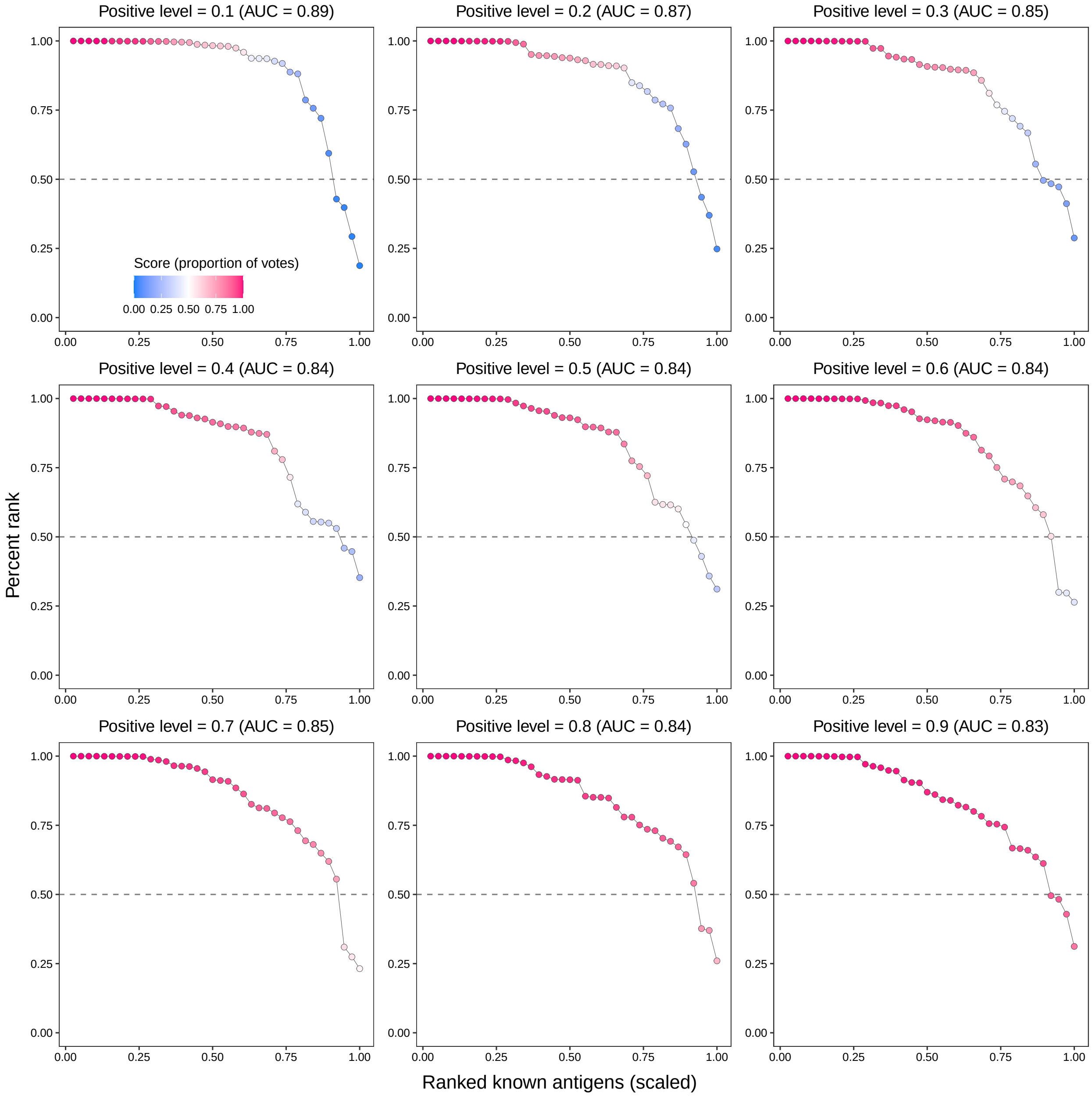

Supplementary Fig. 2 | Evaluation of known antigen predictions of the *P. vivax* model. Subplots of known antigen predictions from *P. vivax* models trained with different positive level (model hyper-parameter) settings, ranging from 0.1 to 0.9. Dots represent the 38 *P. vivax* known antigens, and the x-axes show the scaled ranks of these known antigens. The percentile ranks (the higher the better) calculated across all *P. vivax* proteins are indicated by the y-axes. The grey dashed lines show the percentile rank of 0.5, and the gradient colors represent probability scores (proportion of votes), with darker magenta color showing higher scores and darker blue showing lower scores. The areas under the curves (AUC) are noted in the subplot titles.

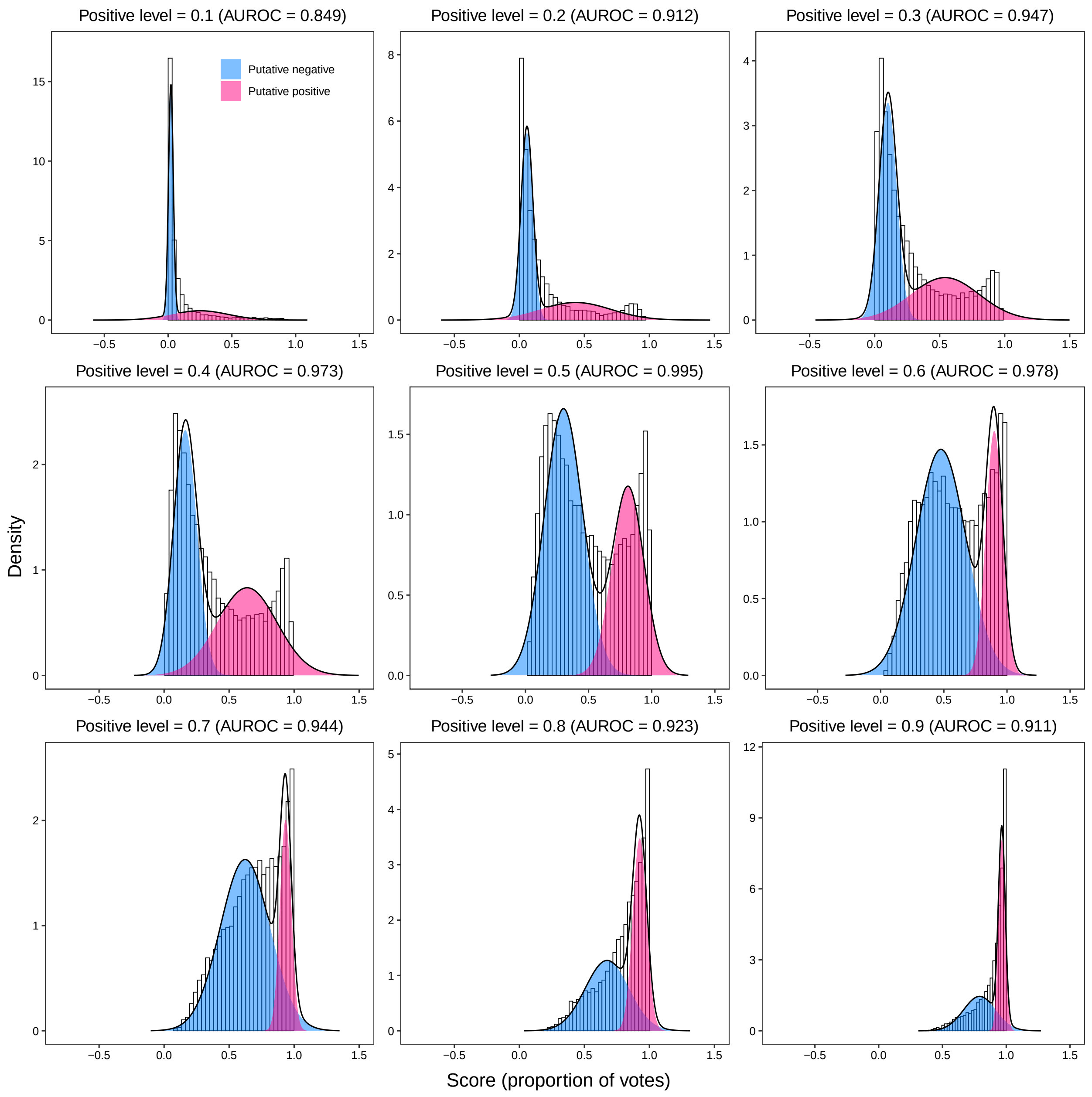

Supplementary Fig. 3 | Hyper-parameter tuning for PURF model trained on the combined data set. Subplots displaying distributions of probability scores (proportion of votes) of unlabeled proteins predicted by combined models trained on varying positive levels (hyper-parameters). The score distributions were modeled using a two-component Gaussian mixture to estimate the putative positive (magenta) and negative (blue) distributions. Receiver operating characteristic curves (ROC) were generated based on these estimated distributions. The areas under the receiver operating characteristic curves (AUROC) are indicated in the subplot titles.

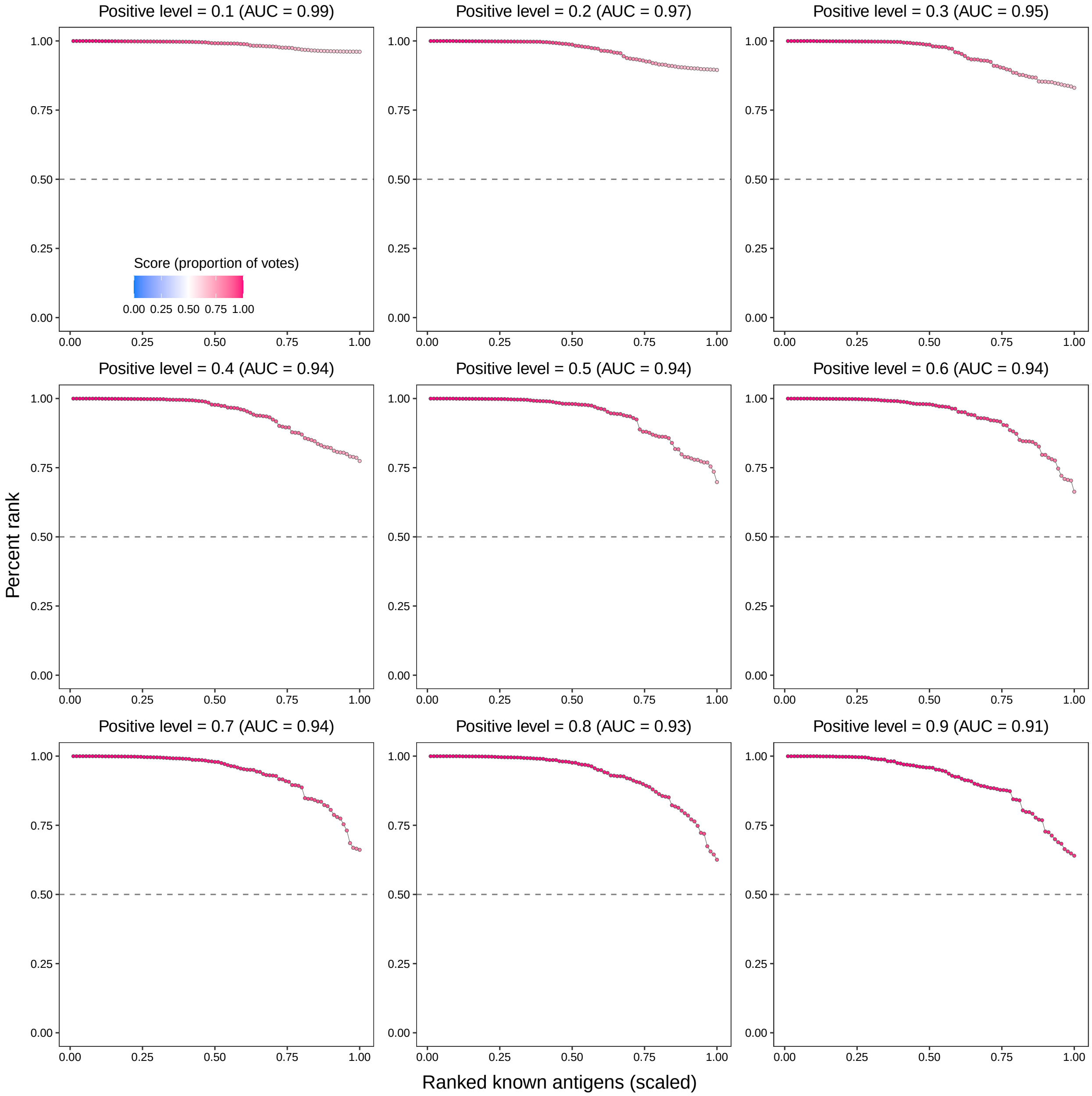

Supplementary Fig. 4 | Evaluation of known antigen predictions of the combined model. The subplots show percentile ranks of known antigen predictions of combined models trained with different positive level (model hyper-parameter) settings from 0.1 to 0.9. The x-axes show scaled ranks of the 90 known antigens from both *Plasmodium* species, and the y-axes indicate percentile ranks (the higher the better) of the known antigens across all proteins from both species. The grey dashed lines show the percentile rank of 0.5. Gradient colors convey probability scores (proportion of votes), with higher scores represented by darker magenta color and lower scores by darker blue color. The areas under the curves (AUC) are noted in the subplot titles.

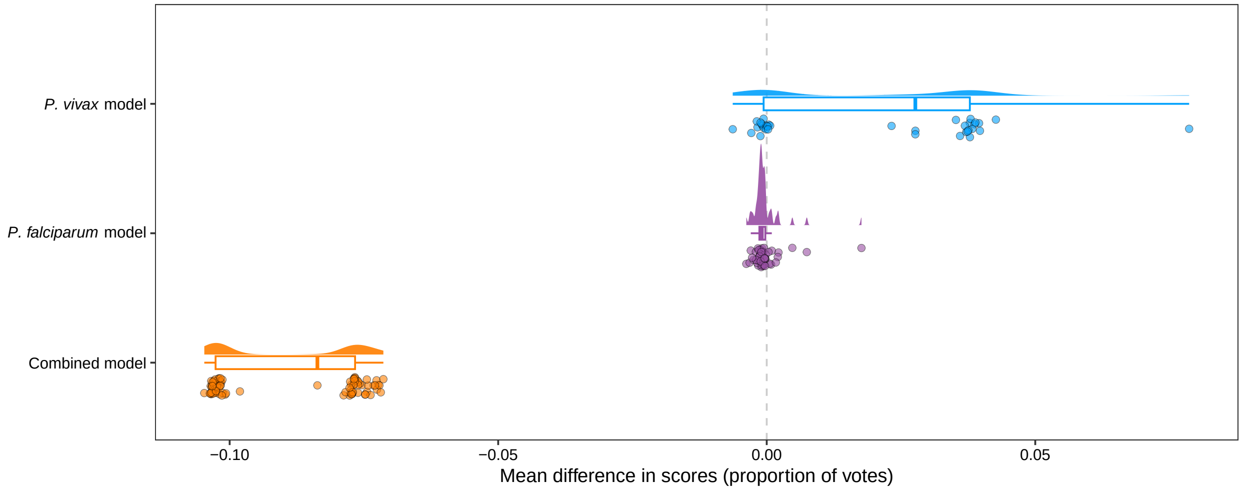

Supplementary Fig. 5 | Validation of PURF models. Mean differences in probability scores (proportion of votes) of the known antigens after removing the label of one of the known antigens in the input data sets for training the *P. vivax* (blue), *P. falciparum* (purple), and combined (orange) models. Boxplots show the median with first and third quartiles, and the whiskers display the extension of the 1.5 interquartile range from the first and third quartiles. The grey dashed line indicates zero mean difference in scores.

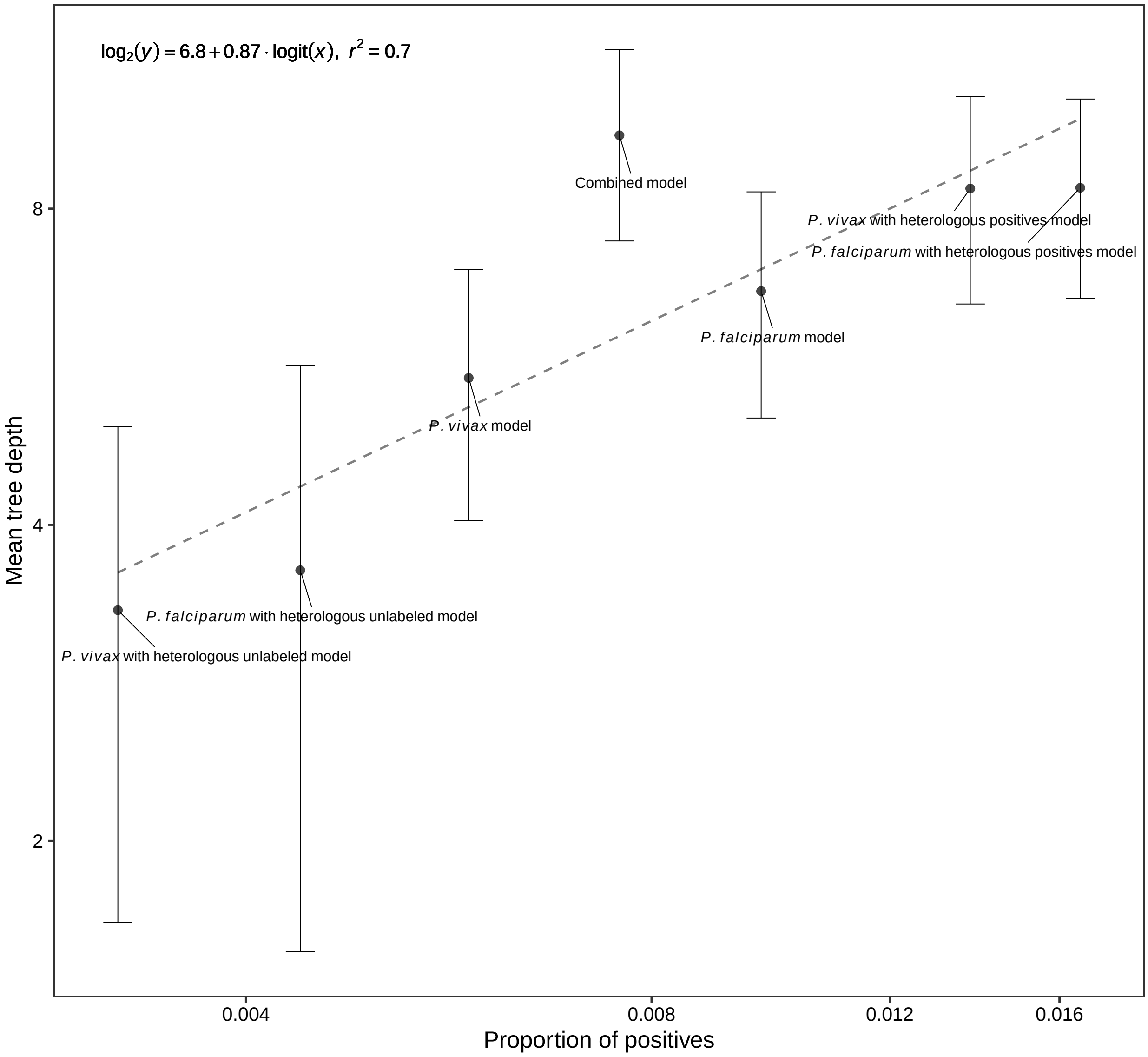

Supplementary Fig. 6 | Relationship between proportion of labeled positives in the data set and mean tree depth in the PURF model. The x-axis is logit-transformed and indicates the proportion of labeled positives in the data set. The y-axis is log_2-transformed and shows the mean depth across all trees in the PURF model. Dots represent PURF models ($n = 7$) with different combinations of autologous and heterologous data, and the model names are noted. Data are shown as mean ± SD. The grey dashed trend line conveys the linear regression model, where the formula and adjusted R^2 are indicated on the upper left corner. The p-value associated with the F-statistic of the linear regression is 0.012.

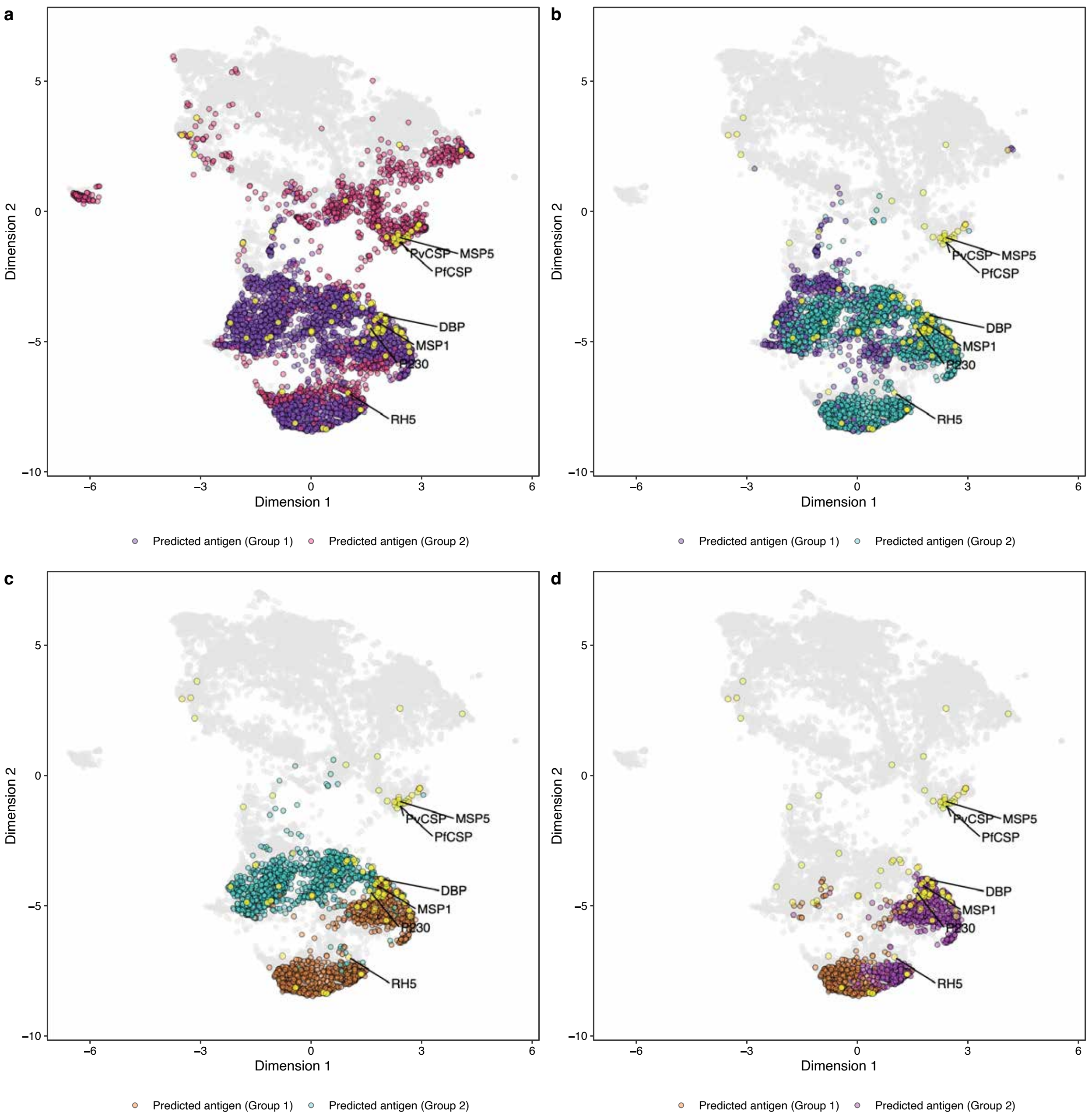

Supplementary Fig. 7 | Visualization of hierarchical clustering dendrogram investigation. Uniform manifold approximation and projection (UAMP) plots showing the iterative investigation of the dendrogram computed from the Euclidean distance matrix of predicted antigens derived from the combined PURF model. **a** The dendrogram was first cut into two groups, where the purple dots and pink dots show group 1 and group 2 predicted antigens, respectively. **b** Group 1 from **(a)** with a higher mean probability score was further divided into two groups, where purple and green dots indicate the new group 1 and group 2 predicted antigens, respectively. **c** The iteration continued and group 2 from **(b)** was selected because of the higher mean probability score, and further divided into another two groups separately represented by green and orange dots. **d** Group 1 from **(c)** was selected and another two groups of predicted antigens were generated based on the sub-dendrogram structure. The respective dot colors for the new group 1 and group 2 are orange and purple. Yellow dots are known antigens from both *Plasmodium* species and the reference antigens are noted by text. Grey dots represent other unlabeled proteins.

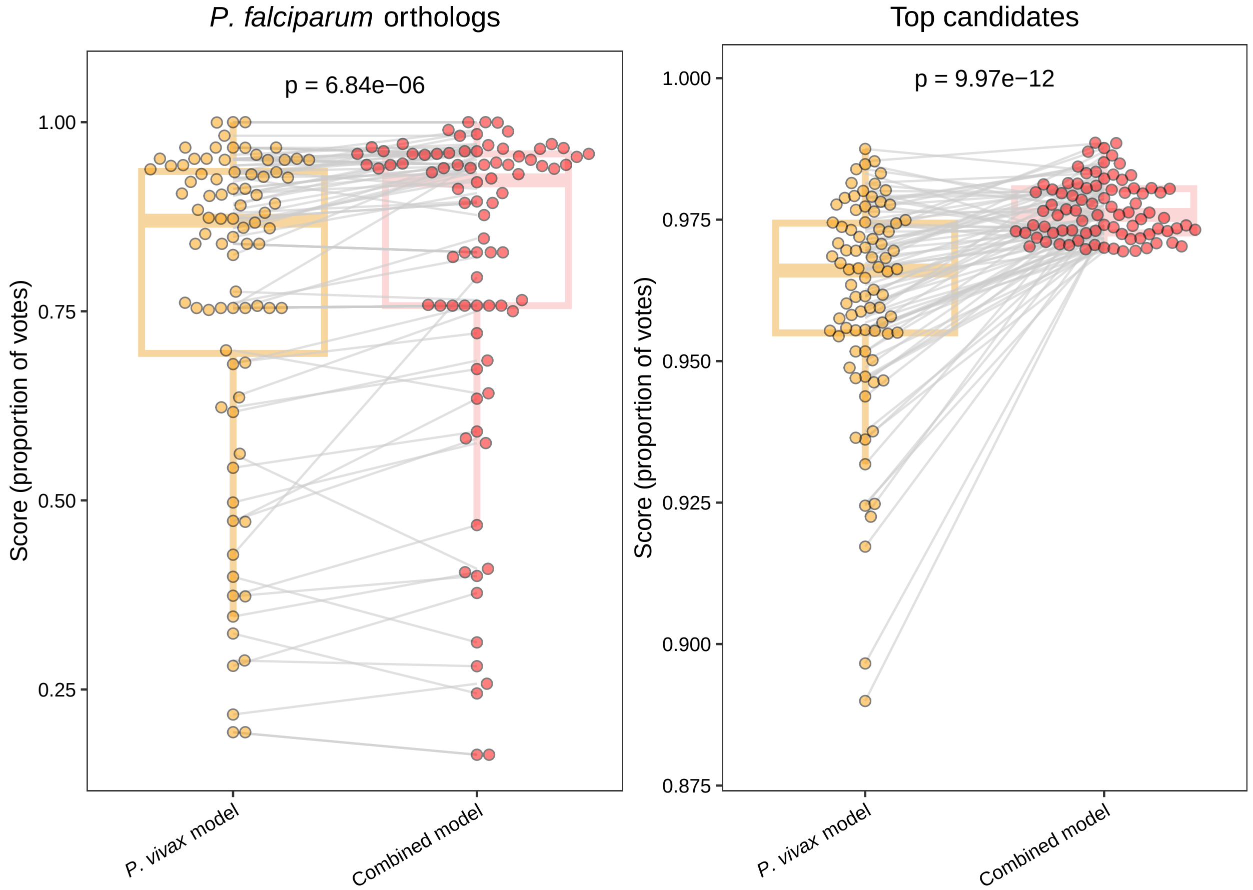

Supplementary Fig. 8 | Probability scores of *P. falciparum* known antigen orthologs and top candiates from the combined model. Comparison of scores predicted by *P. vivax* and combined models for the *P. falciparum* known antigen orthologs (yellow points, $n = 84$) and top candidate antigens from the combined model (red points, $n = 84$). Boxplots indicate the medians with the first and third quartiles. The lower and upper whiskers show 1.5-times the interquartile range extended from the first and third quartiles, respectively. Grey lines connect pairs of the same proteins with different prediction scores. Adjusted p-values from two-sided pairwise Mann–Whitney tests are shown on the top of the plots.

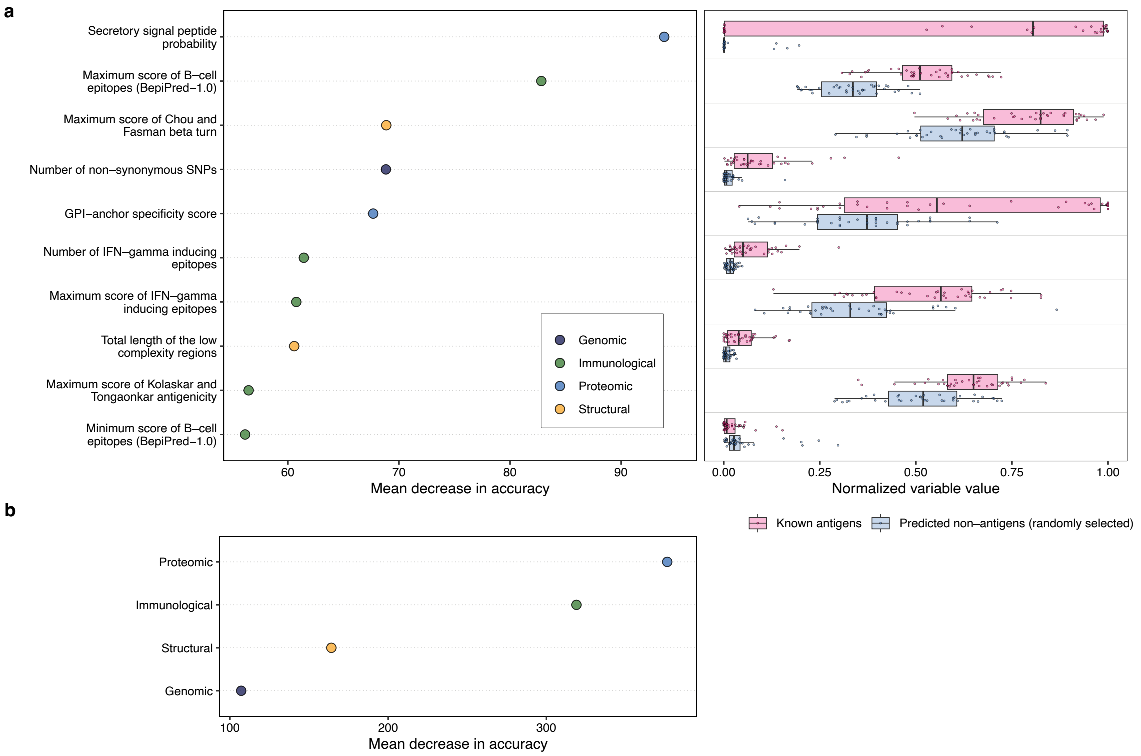

Supplementary Fig. 9 | Variable importance for the *P. vivax* model. a Top 10 important variables are shown on the left panel and categorized into genomic (dark blue), immunological (green), proteomic (blue), and structural (amber) variables. The x-axis indicate importance values in terms of mean decrease in prediction accuracy (scaled by the standard error) of the known antigens after variable permutation. The right panel displays comparisons of normalized variables values between the 38 known antigens (magenta dots) and the same number of randomly selected predicted non-antigens (blue dots). Boxplots show median with first and third quartiles, and the whiskers are the 1.5 interquartile range extended from the first and third quartiles. Two sided Mann–Whitney tests were computed, and the p-values were adjusted using the Benjamini–Hochberg procedure and noted on the right of the panel. **b** The importance of grouped variables by data types, where variables in the same variable data type were permuted together to calculate the mean decrease in accuracy of the known antigens.

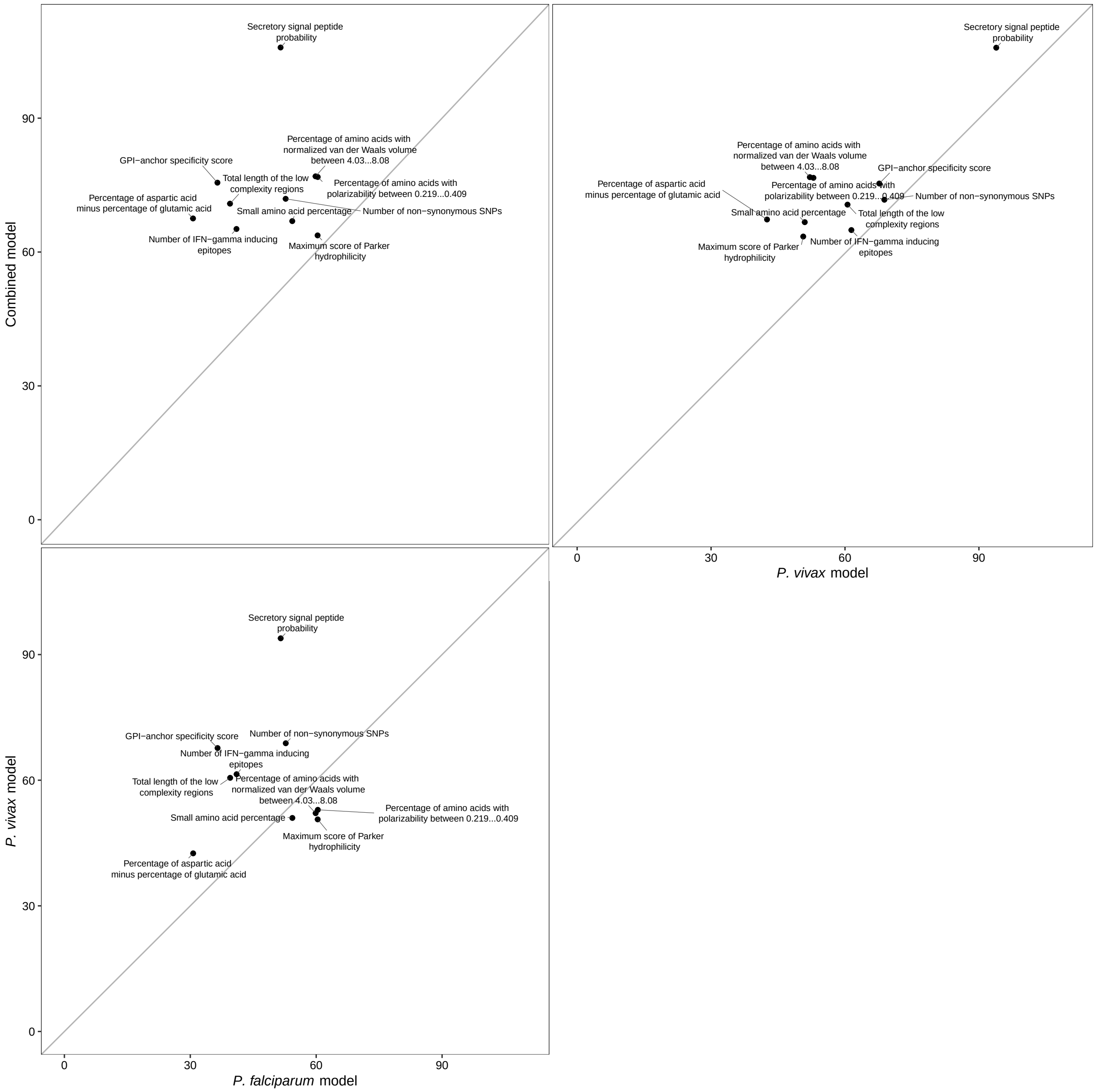

Supplementary Fig. 10 | Comparison of variable importance values between PURF models. Top 10 important variables were identified from the combined models, and the corresponding variable importance values are presented and compared for the combined, *P. vivax*, and *P. falciparum* model. Variable names are noted by text. The grey diagonal lines indicate where the importance values from both compared models are the same.

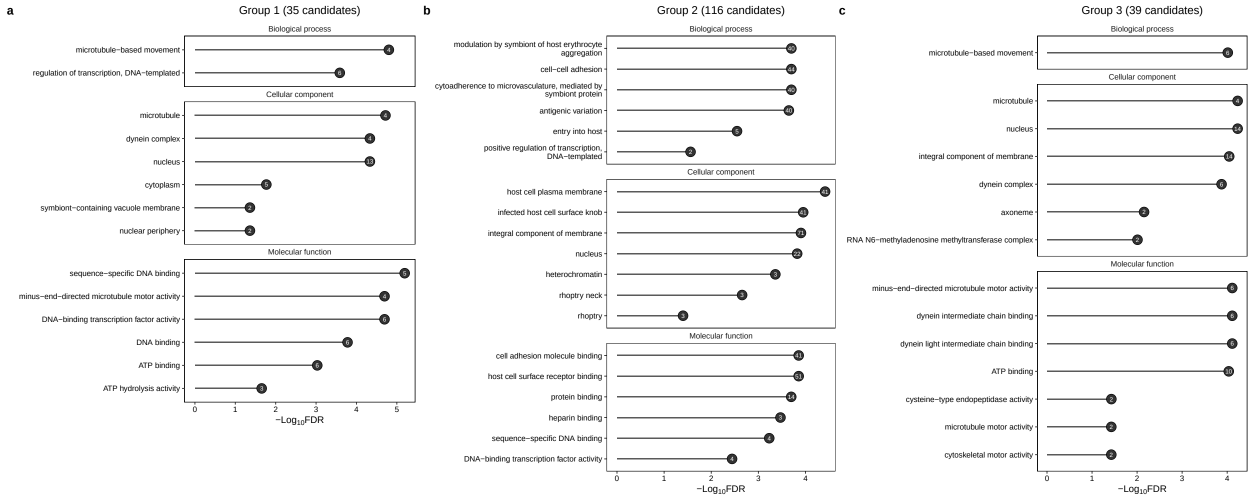

Supplementary Fig. 11 | Gene ontology (GO) enrichment analysis of candidate antigen groups. Plots showing enriched GO terms for group 1 (**a**), group 2 (**b**), and group 3 (**c**) candidate antigen genes, compared to the combined background proteomes of *P. vivax and P. falciparum.* GO terms with false discovery rate (FDR) < 0.05 are shown on the y-axes and categorized into biological process, cellular component, and molecular function. The x-axes show $-log_{10}$FDR, which are indicated by the grey bars of the corresponding GO terms. The values in the black dots indicate the number of antigen genes associated with the GO terms.

3 Supplementary Tables

Supplementary Table 1 | Description of the 38 *P. vivax* known antigens.

Protein ID	Antigen type	Gene produce	Source
PVP01_0623800.1-p1	Reference antigen	duffy binding protein	Reference
PVP01_0728900.1-p1	Reference antigen	merozoite surface protein 1	Reference
PVP01_0835600.1-p1	Reference antigen	circumsporozoite (CS) protein	Reference
PVP01_0215600.1-p1	Known antigen	dynein heavy chain, putative	IEDB
PVP01_0932400.1-p1	Known antigen	kelch domain-containing protein, putative	IEDB
PVP01_1240600.1-p1	Known antigen	conserved Plasmodium protein, unknown function	IEDB
PVP01_0813700.1-p1	Known antigen	conserved Plasmodium protein, unknown function	IEDB
PVP01_1453900.1-p1	Known antigen	peptidase, putative	IEDB
PVP01_0106300.1-p1	Known antigen	EF hand domain-containing protein, putative	IEDB
PVP01_0106200.1-p1	Known antigen	importin-7, putative	IEDB
PVP01_0829000.1-p1	Known antigen	valine–tRNA ligase, putative	IEDB
PVP01_1306000.1-p1	Known antigen	conserved Plasmodium protein, unknown function	IEDB
PVP01_0307400.1-p1	Known antigen	conserved Plasmodium protein, unknown function	IEDB
PVP01_0923000.1-p1	Known antigen	conserved Plasmodium protein, unknown function	IEDB
PVP01_0522300.1-p1	Known antigen	PhIL1-interacting candidate PIC3, putative	IEDB
PVP01_1412900.1-p1	Known antigen	V-type proton ATPase catalytic subunit A, putative	IEDB
PVP01_1255000.1-p1	Known antigen	rhoptry neck protein 2	Intersect
PVP01_1446800.1-p1	Known antigen	merozoite surface protein 9	Intersect
PVP01_1136400.1-p1	Known antigen	6-cysteine protein P12	Intersect
PVP01_0934200.1-p1	Known antigen	apical membrane antigen 1	Intersect
PVP01_0701200.1-p1	Known antigen	reticulocyte binding protein 1a	Literature
PVP01_0418300.1-p1	Known antigen	merozoite surface protein 4	Literature
PVP01_0418400.1-p1	Known antigen	merozoite surface protein 5	Literature
PVP01_0616000.1-p1	Known antigen	ookinete surface protein P28, putative	Literature
PVP01_1030900.1-p1	Known antigen	merozoite surface protein 3	Literature
PVP01_0317900.1-p1	Known antigen	rhoptry-associated leucine zipper-like protein 1	Literature
PVP01_0616100.1-p1	Known antigen	ookinete surface protein P25	Literature
PVP01_0102300.1-p1	Known antigen	duffy binding protein 2	Literature
PVP01_1129100.1-p1	Known antigen	merozoite surface protein 10, putative	Literature
PVP01_0118900.1-p1	Known antigen	von Willebrand factor A domain-related protein, putative	Literature
PVP01_0529100.1-p1	Known antigen	apical merozoite protein	Literature
PVP01_0205500.1-p1	Known antigen	cold-shock protein, putative	Literature
PVP01_0202200.1-p1	Known antigen	tryptophan-rich protein	Literature
PVP01_0304300.1-p1	Known antigen	6-cysteine protein P41	Literature
PVP01_0210500.1-p1	Known antigen	StAR-related lipid transfer protein, putative	Literature
PVP01_1026000.1-p1	Known antigen	6-cysteine protein P38, putative	Literature
PVP01_0532400.1-p1	Known antigen	cysteine-rich protective antigen	Literature
PVP01_0707700.1-p1	Known antigen	GDP-fucose protein O-fucosyltransferase 2	Literature

Supplementary Table 2 | Associations between *Plasmodium* species and antigen predictions from models trained on different combinations of autologous and heterologous data (CI: confidence interval)

**PURF model**	**Cramér's V**	χ^2 **test *p*-value**
Combined	0.08 (95% CI: 0.06, 0.10)	4.95×10^{-19}
P. vivax	0.10 (95% CI: 0.08, 0.12)	3.52×10^{-28}
P. falciparum	0.06 (95% CI: 0.04, 0.08)	7.08×10^{-12}
P. vivax with heterologous positives	0.61 (95% CI: 0.60, 0.62)	~ 0
P. falciparum with heterologous positives	0.62 (95% CI: 0.60, 0.63)	~ 0
P. vivax with heterologous unlabeled	0.80 (95% CI: 0.79, 0.81)	~ 0
P. falciparum with heterologous unlabeled	0.96 (95% CI: 0.95, 0.96)	~ 0

p-values $< 2.225074 \times 10^{-308}$ are reported as ~ 0.
